# Dot1 histone methyltransferases share a distributive mechanism but have highly diverged catalytic properties

**DOI:** 10.1038/srep09824

**Published:** 2015-05-12

**Authors:** Iris J. E. Stulemeijer, Dirk De Vos, Kirsten van Harten, Onkar K. Joshi, Olga Blomberg, Tibor van Welsem, Marit Terweij, Hanneke Vlaming, Erik L. de Graaf, A. F. Maarten Altelaar, Barbara M. Bakker, Fred van Leeuwen

**Affiliations:** 1 Division of Gene Regulation, Netherlands Cancer Institute, Amsterdam, 1066 CX, The Netherlands; 2 Department of Biology, University of Antwerp, Antwerp, 2020, Belgium; 3 Biomolecular Mass Spectrometry and Proteomics Group, The Netherlands Proteomics Centre, Utrecht University, Utrecht, 3584 CH, The Netherlands; 4 Department of Pediatrics, Systems Biology Centre for Energy Metabolism and Ageing, Center for Liver, Digestive and Metabolic Diseases, University of Groningen, University Medical Center Groningen, Groningen, 9713 GZ, The Netherlands

## Abstract

The conserved histone methyltransferase Dot1 establishes an H3K79 methylation pattern consisting of mono-, di- and trimethylation states on histone H3 via a distributive mechanism. This mechanism has been shown to be important for the regulation of the different H3K79 methylation states in yeast. Dot1 enzymes in yeast, *Trypanosoma brucei* (TbDot1A and TbDot1B, which methylate H3K76) and human (hDot1L) generate very divergent methylation patterns. To understand how these species-specific methylation patterns are generated, the methylation output of the Dot1 enzymes was compared by expressing them in yeast at various expression levels. Computational simulations based on these data showed that the Dot1 enzymes have highly distinct catalytic properties, but share a distributive mechanism. The mechanism of methylation and the distinct rate constants have implications for the regulation of H3K79/K76 methylation. A mathematical model of H3K76 methylation during the trypanosome cell cycle suggests that temporally-regulated consecutive action of TbDot1A and TbDot1B is required for the observed regulation of H3K76 methylation states.

Histones are heavily modified by histone modifiers, which can influence the packaging, accessibility, and usage of the genome. One of these histone modifiers is the evolutionary conserved enzyme Dot1, which establishes a methylation pattern of mono-, di- and trimethylation on histone H3 on lysine 79 (H3K79)^1^. Dot1 has been implicated in a range of key cellular processes^2^,^3^,^4^,^5^. It is involved in gene regulation^6^,^7^,^8^, DNA damage repair and response^9^,^10^, the pachytene checkpoint in meiosis^11^ and cell cycle regulation^12^,^13^. In addition, it stimulates cell proliferation^14^ and survival of MLL-rearranged leukemic lymphocytes and breast cancer cells^15^,^16^,^17^. Given the importance of Dot1 proteins in disease, Dot1 proteins are emerging as appealing candidates for therapeutic intervention strategies in humans and trypanosomes^5^. Recently, several inhibitors of human Dot1-like protein (hDot1L) have been developed and hDot1L inhibitors are currently being tested in clinical trials as epigenetic treatment for MLL-rearranged leukemia^18^,^19^. Therefore, there is a broad interest in understanding the catalytic mechanism of Dot1L and mechanisms that regulate this enzyme and the methylation patterns it generates^3^,^20^,^21^,^22^,^23^,^24^,^25^,^26^,^27^,^28^,^29^,^30^,^31^,^32^. Our previous studies have shown that yeast Dot1 is a histone methyltransferase without a SET domain and with a non-processive (distributive) kinetic mechanism: it establishes mono, di- and trimethylation (H3K79me1, -me2 and -me3) on H3K79 via repetitive rounds of binding to and dissociation from histone H3^22^. This implies that a released H3K79 mono- or dimethylation state is not necessarily the final methylation state and that the generation of a higher methylation state depends on the previous lower one. Most other histone methyltransferases contain a conserved SET domain and generate multiple methylation states on their substrates in a processive manner^21^,^33^,^34^. Changes in the activity or expression of the distributive Dot1 enzyme do not affect all H3K79 methylation states equally but lead to characteristic changes in the relative amount of each state. Indeed, partial Dot1 inhibition does not result in an overall reduction of the number of methylated H3K79 residues, but leads to a severe loss of H3K79me3 and a gain of H3K79me1^21^,^22^. Thus, understanding the mode of action of Dot1 has helped to understand the mechanisms of regulation of H3K79 methylation. One consequence of the distributive mechanism in combination with the absence of a known H3K79 demethylase is that H3K79me3 accumulates on ageing histones, which makes H3K79 methylation act as a molecular timer in the cell^21^,^35^.

The Dot1 catalytic domain and the substrate lysine on histone H3 on the nucleosome core are highly conserved^22^,^36^. Interestingly, the global methylation patterns vary substantially between different organisms. Whereas in yeast the majority of H3 is methylated (~90%) and mainly in the H3K79me3 state^1^,^22^, in human and mouse cells only a small subset of H3 is methylated, predominantly in the form of H3K79me1^37^. In the unicellular eukaryotic parasite *Trypanosoma brucei*, the causative agent of African sleeping sickness, two Dot1 enzymes TbDot1A and TbDot1B are responsible for methylation of H3K76, the *Trypanosoma* counterpart of H3K79 in yeast and larger eukaryotes. Interestingly, in trypanosomes the majority of H3K76 is di- or trimethylated by TbDot1A and TbDot1B, respectively^12^,^23^,^38^.

In trypanosomes, TbDot1B is required for proper silencing of telomeric VSG expression sites, a process crucial for antigenic variation of these parasites^4^,^39^,^40^, normal cell cycle progression, and differentiation of the bloodstream form to the procyclic insect stage^12^. Depletion of TbDot1A and loss of H3K76me2 abolished DNA replication while TbDot1A overexpression resulted in continuous DNA replication^38^. In human cells, Dot1L may also have a role in DNA replication because H3K79me2 has been found to associate with origins of replication to help limit DNA replication once per cell cycle^41^.

Here we determined the intrinsic enzymatic properties of four Dot1 enzymes that generate highly divergent H3K79/76 methylation patterns, namely hDot1L, TbDot1A, TbDot1B and yeast Dot1 (yDot1). To unravel their mechanism of methylation and apparent catalytic rate constants the enzymes were studied by heterologous expression in yeast, allowing direct comparison under identical physiological conditions. By obtaining quantitative measurements of H3K79 methylation patterns and combining it with computational simulations, we found that the different Dot1 enzymes share a distributive mechanism, but show highly divergent catalytic properties reflected by distinctive rate constants.

## Methods

### Yeast strains and plasmids

Strains and plasmids used in this study are specified in Supplemental Table SII. Strains were derived from S288C strains BY4741 and Y7092^42^,^43^. All integrations and deletions were made using homologous recombination. To express TbDot1A, TbDot1B or hDot1L from the endogenous yeast *DOT1* locus, first *DOT1* was replaced by a *URA3* selection cassette using the 5’ and 3’UTR flanking sides. Subsequently, the *URA3* cassette was replaced by ORF sequences amplified by PCR from pCJ49 (TbDot1A), pFF019 (TbDot1B) and pFvl925 (hDot1L). To obtain strains expressing different concentrations of Dot1 enzyme, different promoters were integrated ~100 base pairs upstream of the *DOT1* gene (leaving part of the 5’UTR), or immediately upstream of the START codon. Promoter sequences were amplified from pYM-N6 (NatMX-*ADH*pr), PYM-N14 (NatMX-*GPD*pr), pYM-N18 (NatMX-*TEF*pr). To generate NKI6123, the *TbDot1A* gene was replaced by *URA3* from pRS306 in NKI6099 (NKI6107). Subsequently, *URA3* was replaced by a NatMX-*ADH*pr-*hDot1L*1-430 PCR product (NKI6120) and a 9xMyc-KanMX was added (NKI6126A) as described above. Finally, the *TRP1*-*GAL1*pr-spacer-NLS-3xFLAG was PCR amplified from pIS013 and integrated in frame with *hDot1L* in NKI6126A (NKI6123). The 9xMyc-tagging cassette from pYM20 (9xMyc-HphNt1) was introduced distal of the N-terminal part of the gene, thereby disrupting the STOP codon and allowing expression of a 9xMyc-tagged Dot1 protein. *DOT1* was deleted using primers DOT1KO1 and DOT1KO2 together with plasmid pRS306 (NKI6069) or pRS400 (NKI4506). pIS013 was generated by cloning a 200 bp fragment consisting of NLS-3xFLAG from pUC57-NLSTAG3e into pTCG using *Xho*I and *Bam*HI. Subsequently, *hDot1L* was integrated using *Eco*RI and *Sal*I sites to allow expression of NLS-3xFLAG-*hDot1L*1-430 (pTW125).

### H3K79 methylation pattern analysis

For analysis of H3K79 methylation and Dot1 protein expression cells were harvested in mid-log phase as described previously and in the Supplemental Methods. Quantitative western blot analysis was performed using the LI-COR Odyssey IRDye® IR imager (Biosciences) and the Odyssey LI-COR software. For details about the normalization of the H3K79 methylation signals based on the linearity of the antibodies, see the Supplemental Methods. Histone H3 purification for mass spectrometry and determination of growth rates was performed as described previously^22^. Data were obtained of at least two replicates.

### Computational simulations of H3K79 methylation patterns

A previously described model^21^ was used to simulate H3K79 methylation patterns and estimate *in vivo* Dot1 rate constants. To simulate time-dependent changes in H3K76 methylation during the course of the cell cycle in trypanosomes, the distributive model for Dot1 methylation was integrated with histone dynamics during the cell cycle (see Supplemental Methods).

### RITE assay

**Figure 1:**
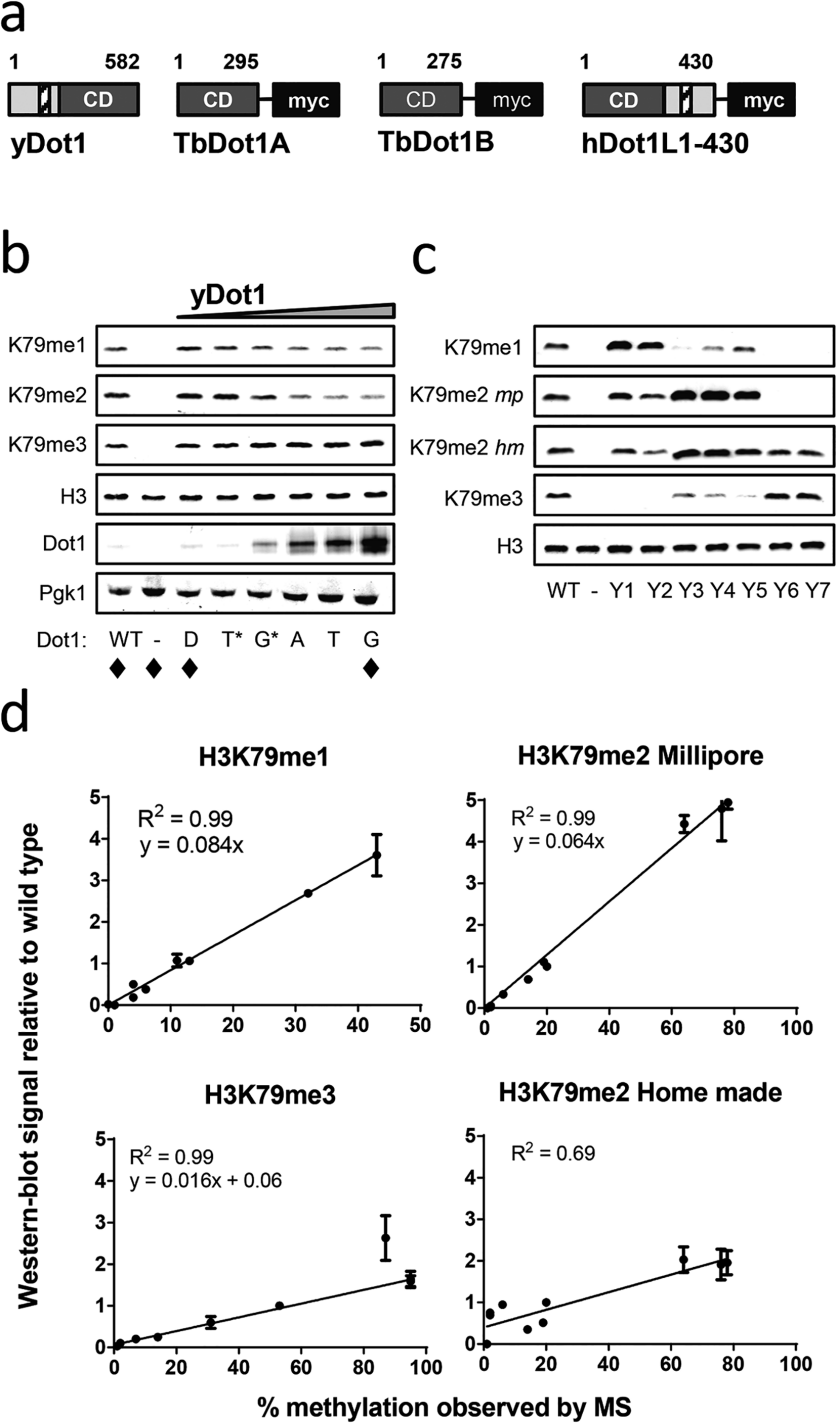
Quantitative H3K79 methylation analysis on a series of Dot1 alleles. A) Schematic overview of yeast Dot1, trypanosome Dot1A and Dot1B, and human Dot1L. The enzymes share a conserved catalytic domain (grey box, CD). In addition, yDot1 contains an N-terminal domain with a lysine-rich domain (light grey and white shaded box, respectively). Human Dot1L was expressed with part of its C-terminal domain that has weak similarity with the yDot1 N-terminal domain. B) Western-blot analysis of H3K79 methylation and yDot1 expression using specific antibodies. H3 and Pgk1 were used as loading controls. A wild-type yeast strain (NKI6061; WT) was used as a reference throughout the manuscript; its H3K79 methylation levels were determined by mass spectrometry. A *dot1Δ* (–) was included to determine antibody specificity. Dot1 enzymes were expressed from the following promoters at the endogenous yeast *DOT1* locus: *DOT1*pr (D), *TEF*pr + *yDOT1* 5’UTR (T*), *GPD*pr + *yDOT1* 5’UTR (G*), *ADH*pr (A), *TEF*pr (T) or *GPD*pr (G). H3K79 methylation patterns confirmed by mass spectrometry (MS) are indicated with ♦. C) Western-blot analysis of a series of reference yeast strains (Y1-Y7) containing a range of known H3K79 methylation levels (see below) to determine the linearity of the H3K79me1, -me2 and -me3 home-made (*hm*) antibodies^22^ and the H3K79me2 antibody from Millipore (*mp*). Y1-Y7 refers to strains: NKI6081, NKI6077, NKI6084, NKI6099, NKI6100, NKI6085 and NKI6083. D) Samples described in (C) were quantified using an Odyssey scanner and by MS. To determine the linearity of the H3K79 methylation antibodies, MS data were plotted against the quantified western-blot data and a non-linear regression fit was performed (see Supplemental Methods). The linear function resulting from the fit was used to correct the quantified western-blot data described in this manuscript and to subsequently estimate the unmethylated H3 fraction. No linear fit was obtained for the H3K79me2 home-made antibody; the low correlation between MS and western-blot data of the H3K79me2 home made antibody was caused by cross-reactivity of this antiserum with H3K79me3^22^. Unless stated otherwise, H3K79me2 was quantified using the Millipore antibody, since this showed very little cross-reactivity to H3K79me3.

**Figure 2:**
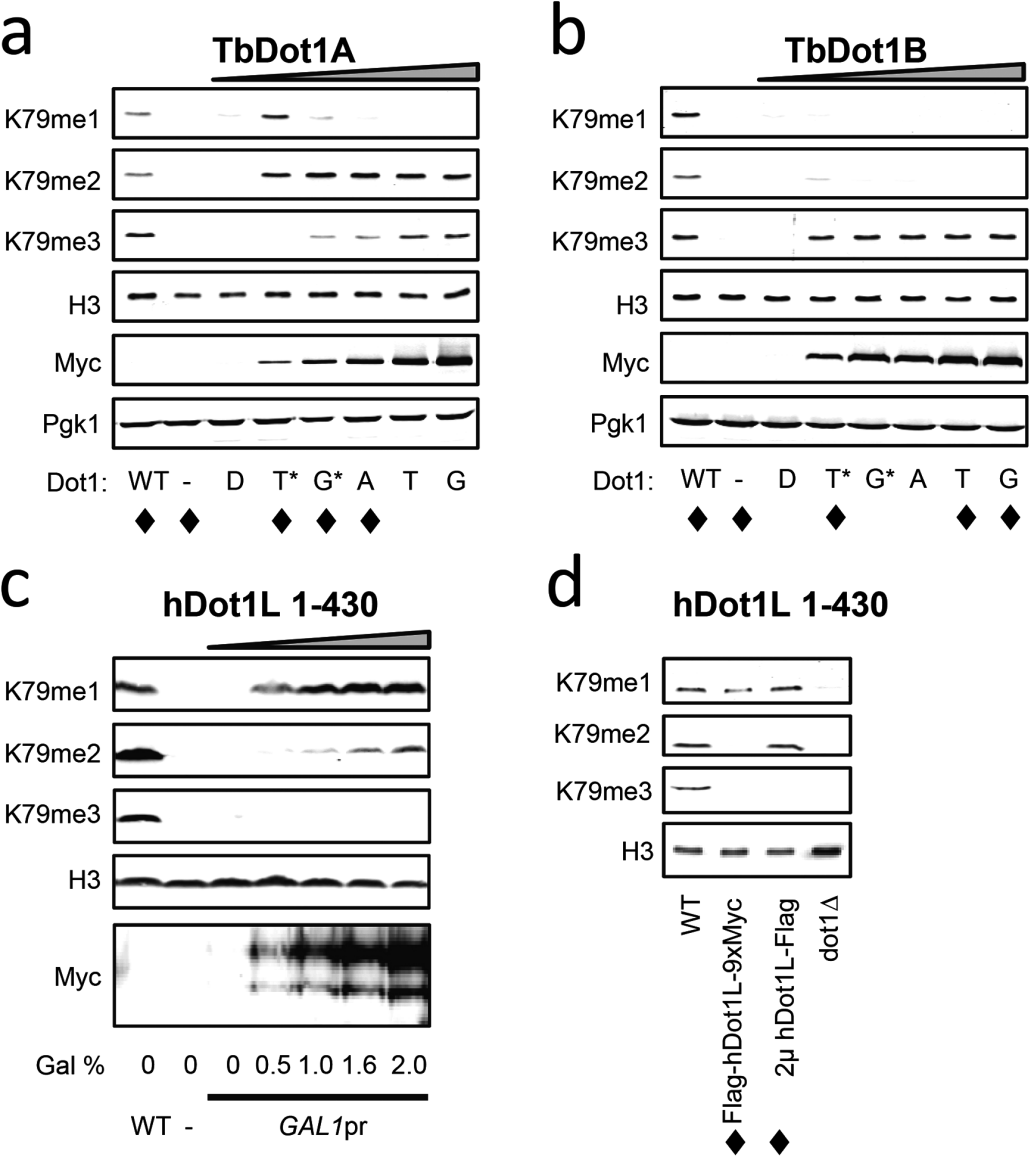
Quantitative assessment of H3K79/K76 methylation by TbDot1A, TbDot1B and hDot1L. Western-blot analysis of H3K79 methylation states generated by TbDot1A (A), TbDot1B (B) and hDot1L (C) upon expression from the *yDOT1* locus in yeast. H3K79 methylation states were determined using specific H3K79 methylation antibodies and the Dot1 enzymes were detected using their Myc and/or Flag tags. H3 and Pgk1 were used as loading controls. H3K79 methylation patterns that were confirmed by mass spectrometry are indicated with ♦. A-B) TbDot1A (A) and TbDot1B (B) enzymes were expressed from the same promoter series as described in Fig. 1B. C) hDot1L was expressed in yeast from the *GAL1* promoter at the endogenous *yDOT1* locus (Flag-hDot1L-9xMyc) upon induction by 2% galactose as the sole carbon source in rich medium. D) hDot1L was expressed in yeast from the *GAL1* promoter at the endogenous *yDOT1* locus (Flag-hDot1L-9xMyc) or at a multi-copy plasmid (2µ-hDot1L-Flag) upon induction by 2% galactose as the sole carbon source in the minimal medium. Human Dot1L protein expression levels are shown in Supplemental Fig. S4.

Recombination Induced Tag Exchange (RITE) strains in which yeast *DOT1* was deleted were transformed with pTW100 (TbDot1A) or pTW098 (TbDot1B), while a *DOT1* wild-type RITE strain was transformed with pFvl261 (empty vector). To track how yDot1, TbDot1A and -B establish H3K79 methylation patterns on newly synthesized histone H3, the RITE assay, which has been described previously^44^,^45^, was slightly modified. Briefly, strains were grown during the full experiment in YC media lacking tryptophan and containing 2% galactose as carbon source. Cells were grown for three days into starvation, after which they were diluted and grown to mid-log phase overnight. Cells were subsequently further diluted to maintain cells in log-phase during the course of the experiment and ß-estradiol was added to induce the recombinant-induced tag exchange from T7 to HAHIS on histone H3. Samples for western blot were taken after each population doubling (1 doubling per 3 hours in this media) and both old (H3-T7) as well as new (H3-HAHIS) histones were detected using the anti-LoxP antibody^44^. H2A (39235, Active Motif) or H2B (39237, Active Motif) detection was used as loading control, while H3K79 methylation patterns were detected using the above described antibodies. H3K79 methylation states on “new” H3-HAHis were quantified and normalized to the average H3K79 methylation pattern (in %) of the 100% new H3-HAHis strain (∞). The average H3K79 methylation pattern of the 100% new H3-HAHis strain on western blot was compared to the steady state H3K79 methylation patterns presented in Figs. 1 and 2. The determined H3K79 methylation levels by MS of the best matching strain was used as a reference for yeast cells expressing yDot1 (*yDOT1*pr-yDot1), TbDot1A (*TEF*pr-TbDot1A) or TbDot1B (*GPD*pr-TbDot1B) (Supplemental Table SIII).

## Results

Here, we analyzed and compared the H3K79 methylation properties of Dot1 enzymes from *Saccharomyces cerevisiae* (yDot1), *Trypanosoma brucei* (TbDot1A and TbDot1B) and human (hDot1L; Fig. 1A). We aimed to express the Dot1 enzymes from the endogenous yDot1 locus in *S. cerevisiae* at different expression levels to determine from the H3K79 methylation patterns whether they act by a processive or distributive mechanism and to determine their catalytic properties. To validate this approach, first yDot1 was expressed from a series of four promoters, some of which were combined with the 5’UTR region of yDot1, which is known to reduce expression from ectopic promoters^46^. This resulted in a series of promoter alleles that showed a wide range of expression levels (Fig. 1B). As expected for yDot1, the H3K79me3 state increased while the H3K79me1 and -me2 states decreased upon a gradual increase of yDot1 protein expression (Fig. 1B), which is a consequence of the distributive mechanism of yDot1. To generate quantitative data for mathematical modeling we analyzed H3K79 methylation patterns relative to wild-type methylation by quantitative immunoblot analysis and analyzed absolute H3K79 methylation patterns by mass spectrometry (MS). The linearity of the antibodies was determined using MS data of a panel of selected yeast strains with distinct H3K79 methylation states (see also below). The H3K79 methylation patterns determined by immunoblots (Fig. 1C and Supplemental Fig. S1) correlated very well with the absolute H3K79 methylation states determined by mass spectrometry (Supplemental Table SI) confirming the linearity of the antibodies (Fig. 1D).

### Dot1 enzymes share a distributive kinetic mechanism but have different apparent rate constants

Next, we used the same promoter series to express TbDot1A and TbDot1B from the yDOT1 locus. This resulted in yeast strains expressing a range of *Trypanosoma* Dot1 protein levels which showed very divergent global H3K79 methylation patterns (Fig. 2A–B). TbDot1A generated a strong prominent H3K79me2 signal at intermediate expression levels. However, at low expression levels H3K79me1 was more abundant, while H3K79me3 was found upon increasing TbDot1A expression (Fig. 2A). TbDot1B almost exclusively generated H3K79me3, also at low expression levels that are comparable to TbDot1A expression (Fig. 2B). H3K79me1 and -me2 were detected only at very low TbDot1B expression levels (see also Supplemental Table SIIIA and additional data in Supplemental Fig. S2). For both TbDot1 enzymes the relative abundance of the three methylation states was variable upon changes in expression level, which is in agreement with the distributive mechanism of action that was recently demonstrated by *in vitro* methyltransferase assays^23^.

We next analyzed hDot1L. Expression of hDot1L1-430 from the promoters described above resulted in very low protein expression for reasons that we do not understand. As an alternative approach, we expressed hDot1L1-430 with a nuclear localization signal (NLS) from a galactose-inducible promoter at the endogenous yDOT1 locus. Despite being expressed at levels higher than endogenous yDot1 (Supplemental Fig. S4) the hDot1L protein showed a very low catalytic activity when compared to the other Dot1 enzymes studied here, resulting in mainly a small amount of H3K79me1. As an additional approach, a galactose-inducible hDot1L allele on a multi-copy plasmid was used to generate higher hDot1L expression levels. Under that condition, H3K79me1 as well as H3K79me2 was generated (Fig. 2D). The change in relative abundance of the H3K79 methylation states – increase in H3K79me2 without increased H3K79me1 (Fig. 2D and Supplemental Fig. S3) – indicates that hDot1L is a distributive enzyme. Taken together, our data suggest that the distributive mechanism is conserved among Dot1 enzymes.

To perform a quantitative and unbiased assessment of catalytic activities of the four histone methyltransferases, mathematical models for processive and distributive enzymes were used. These models simulate histone H3K79 methylation dynamics in the cell to reveal the kinetic mechanism and obtain estimates of the rate constants of the respective methylation steps. Yeast is especially well suited for computational modeling because of the ease of ectopic expression and the availability of a unique data set of endogenous protein copy numbers. Besides experimental H3K79 methylation patterns (determined by western blotting and MS), other data used were Dot1 concentration (determined by western blots with tagged yDot1 as a reference with a known copy number) (Figs. 1B and 2, Supplemental Table SIII), growth rates (Supplemental Table SIII), and the H3 concentration in the cell (http://yeastgenome.org/). Rate constants for the respective methylation steps in this model represent the best estimations of the *k_cat_*/*K_m_* ratio for each methylation action *in vivo*. More complicated enzyme kinetic equations typically have too many parameters to allow a unique fit to the available experimental data. Moreover, the Dot1 substrate S-adenosyl-methionine (AdoMet) was assumed to be constant. The kinetic constants in this study therefore represent apparent rate constants.

**Figure 3:**
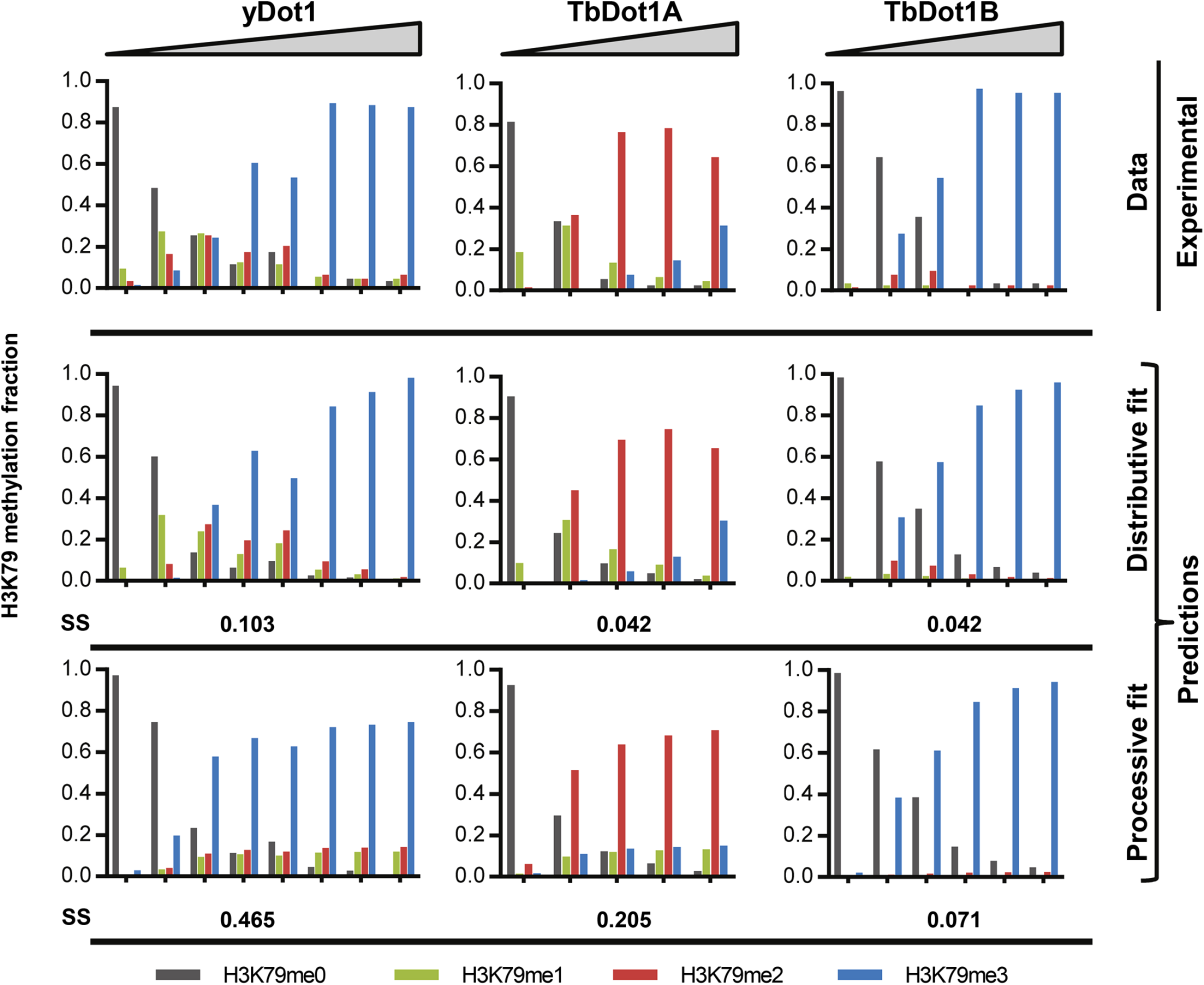
Dot1 enzymes share a distributive mode of action. Experimental data of H3K79 methylation patterns generated by yDot1, TbDot1A, and TbDot1B in yeast and simulations using the models for distributive and processive enzymes as described in De Vos et al (2011)^21^. For each enzyme, the experimental H3K79 methylation pattern data that passed the quality test (presented in Fig. S3 and Supplemental Table SIII) were used to model the kinetic mechanism of the Dot1 enzymes. For yDot1, data from Frederiks et al^22^ were included. A subset of this dataset was confirmed by mass spectrometry analysis (see Supplemental Table SIII for numeric data). Simulations for hDot1L are shown in Supplemental Fig. S4. Each graph represents the H3K79 methylation patterns of yeast strains expressing a Dot1 enzyme at different concentrations. Each tick on the x-axis represents a single yeast strain. Simulations with the distributive model generated a better fit with the experimental data than the simulations with the processive model, as shown by the lower sum of squares values (SS).

This approach previously revealed that yDot1 is a distributive enzyme and that different rate constants are required for the establishment of H3K79me1 (*k_0_*), -me2 (*k_1_*) and -me3 (*k_2_*)^21^. Before applying this approach to H3K79 methylation patterns of the TbDot1 enzymes and hDot1L, the procedure was applied to the extended yDot1 dataset (Supplemental Table SIIIA, Fig. 3 and Supplemental Fig. S3). The analysis confirmed the distributive mechanism of yDot1 and validated the robustness of our experimental system. Furthermore, the model generated similar *in vivo* rate constants as described by De Vos et al^21^ (Table I). Subsequently, we evaluated how well the H3K79 methylation patterns of TbDot1A and -B could be predicted by elementary processive and distributive models. For both enzymes, simulations with the distributive model resulted in a good fit of the experimental data, whereas modeling with the processive model did not fit the experimental data as well (Fig. 3). This confirmed that both TbDot1A and -B are distributive enzymes (Fig. 3).

As expected from the observed different steady-state H3K79 methylation patterns, TbDot1A and -B had different estimated rate constants compared to each other and to yDot1. In trypanosomes, TbDot1A expression has been linked to high H3K76me2. Interestingly, the estimated rate constants suggest that the predominant accumulation of H3K79me2 by TbDot1A in yeast is not the result of high dimethylation efficiency (*k_1_*) but of a ~30 fold reduced trimethylation efficiency (*k_2_*) compared to yDot1, preventing rapid conversion of H3K79me2 into H3K79me3 (Table I and Supplemental Table SIV). TbDot1B expression in trypanosomes is associated with high H3K76me3 levels. Indeed, TbDot1B has a high *k_2_* when compared to yDot1 or TbDot1A; it is able to establish H3K79me3 ~8 times faster than yDot1. Surprisingly however, the *k_1_* value revealed that TbDot1B is also a very efficient dimethylase since it generates H3K79me2 ~15 times more efficiently than yDot1 and TbDot1A (Table I and Supplemental Table SIV). In contrast, even though TbDot1B can mono-, di-, and trimethylate histone H3 without the help of TbDot1A, it is ~2.5 fold slower in the establishment of H3K79me1 (*k_0_*) than yDot1 and TbDot1A. This is not unexpected because in trypanosomes, TbDot1B predominantly acts on H3K76 that was previously di- (or mono-) methylated by TbDot1A^12^. This and possible roles for the strong dimethylase activity of TbDot1B will be discussed below.

Finally, we attempted to model the experimental data of hDot1L. However, a relatively low quality fit was obtained as a result of the extremely high copy number estimation for hDot1L when expressed from the multi-copy plasmid (Supplemental Fig. S4 and Supplemental Table SIII). Likely, the estimated copy number is not representative for the active hDot1L fraction in the cell since the estimated enzyme concentration is ~5 fold higher than the substrate (H3) concentration. Therefore, we could not further model the experimental data of hDot1L to obtain reliable estimates of the rate constants. However, our results do suggest that the hDot1L protein shows a very low catalytic activity compared to yeast or Trypanosoma Dot1 proteins. Together, our findings show that the conserved Dot1 enzymes share a distributive catalytic mechanism with some rate constants remarkably similar, while other constants seem to have diverged resulting in unique H3K79 methylation properties.

### Dot1 rate constants determine the kinetics of H3K79 methylation in the cell

**Figure 4:**
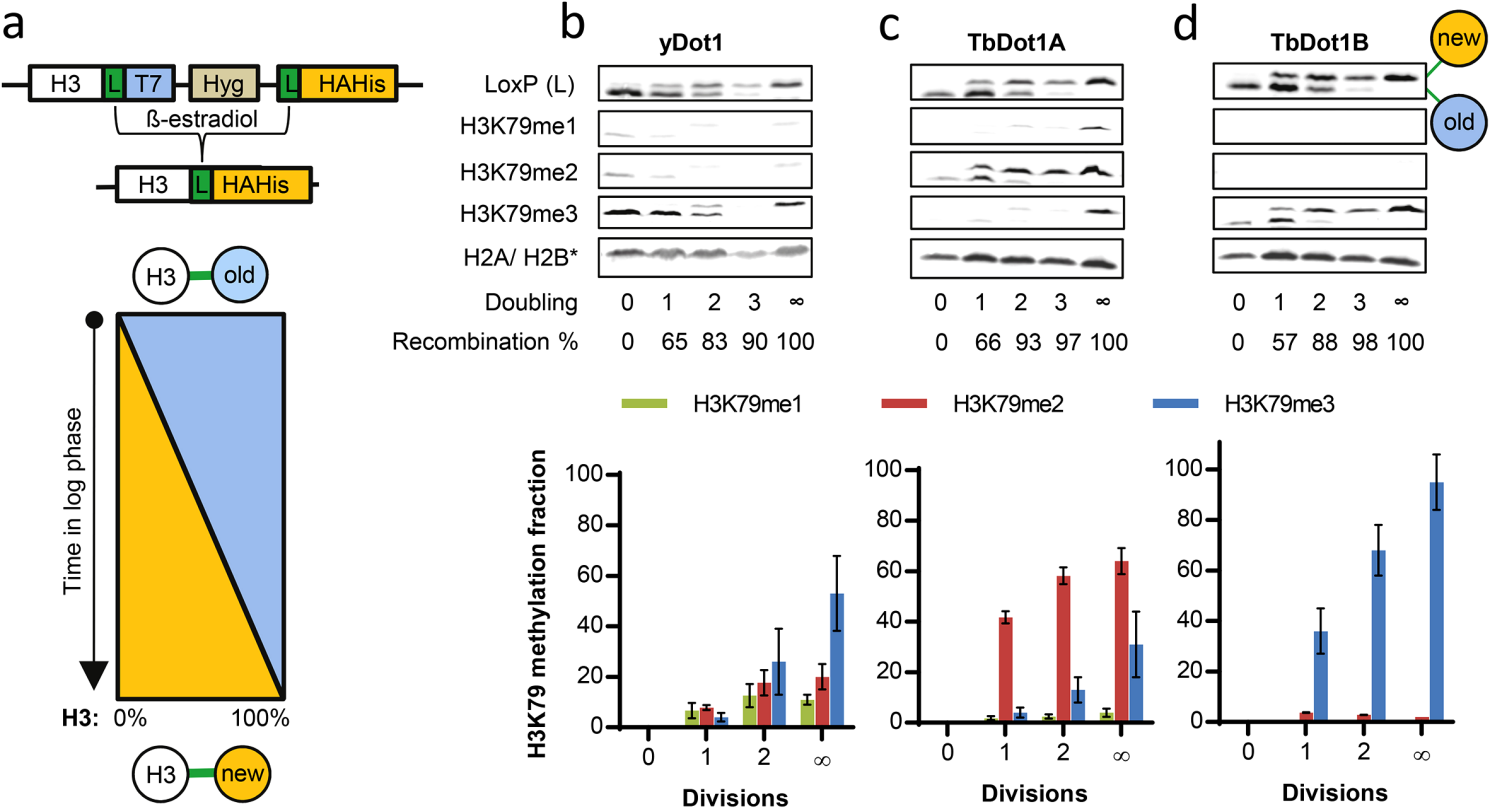
Kinetics of H3K79 methylation of new histones. Dot1 methylation activity on newly synthesized histones was tracked using a modified version of the Recombination-Induced Tag Exchange (RITE) assay in yeast^44^. A) The only gene present encoding for histone H3 (*HHT2*) was tagged with a RITE cassette (LoxP-T7-Hyg-LoxP-HAHis), which resulted in expression of H3 tagged with T7, also referred to as the “old” histone. Addition of β-estradiol in log phase induced recombination between the LoxP sites (and deletion of the T7-hygromycin (HYG) cassette), resulting in the production of “new” H3 tagged with HAHis. Since recombination occurs asynchronously and takes a few hours before it is completed in a population of cells, the percentage of cells in the population that underwent RITE and produced H3-HAHis increased during the course of the experiment. As a consequence, H3 with a new tag represented a mix of histones of different ages; this age distribution changed over time. B-D) Western-blot analysis of H3K79 methylation levels on new (upper band) and old (lower band) histone H3 in the presence of yDot1 (B), TbDot1A-HA-TAP (C) and TbDot1B-HA-TAP (D). Samples were taken upon one, two or three population doublings (Doubling = 1, 2, or 3). As controls, samples were taken immediately after the addition of β-estradiol (100% “old”, Doubling = 0), and from strains that expressed only the H3-HAHis (100% “new”, ∞). Antibodies against H2A (yDot1) or H2B (TbDot1A and -B) were used as loading controls. The percentage of recombination was determined for each sample at the different time points. The graphs represent the average H3K79 methylation levels on newly synthesized histone H3 of which at least two replicates were available. H3K79 methylation states (relative to H2A/H2B and normalized to the new H3 signal of the LoxP blot) were converted into estimated absolute methylation levels by using as a reference the 100% new strain (∞; see Supplemental Fig. S5).

To examine how the apparent rate constants impact on the kinetics of H3K79 methylation in the cell, the accumulation of H3K79 methylation states was studied on newly synthesized unmodified histone H3 using a variant of the Recombination-Induced-Tag-Exchange (RITE) system^44^. Briefly, in log-phase cells, induction of Cre-recombinase activity resulted in a genetic switch from an old epitope-tag on histone H3 to a new tag (Fig. 4A). Using this tag-switch system, newly synthesized histone H3 molecules have a different tag than the old H3 molecules already present in the cell. The two H3 species differ in size and were simultaneously detected by an antibody against the common spacer peptide (LoxP). In the experimental set-up used here, the percentage of cells in the population that performed Cre-mediated recombination and produces ‘new’ H3-HAHis increases during the course of the experiment. As a consequence, H3 with a new tag represents a mix of histones of different ages. However, the average age of the ‘new’ H3-HAHis histones increases over time. The establishment of H3K79 methylation on new histones (upper band) was followed using H3K79 methylation antibodies (Figs. 4B-D and Supplemental Fig. S5). In the strain constitutively expressing yDot1, new histone H3 proteins initially contained H3K79me1 and -me2 but low H3K79me3 levels (e.g. see the 2-doubling time-point where old and new histone H3 are present at similar levels). The H3K79me3 levels increased at later time points (Fig. 4B). This is in agreement with the expectation for a distributive enzyme that new histone H3 initially acquires H3K79me1, which is then converted into H3K79me2 and H3K79me3 (given that for yDot1 *k_0_* > *k_1_* > *k_2_*).

The TbDot1A and -B enzymes were constitutively expressed from a plasmid in a *dot1Δ* RITE strain. Expression of TbDot1A or –B did not affect the kinetics of Cre recombination mediated genetic tag switch (Fig. 4B–D). In the presence of TbDot1A, H3K79me2 signals were rapidly detected on new histone H3, while H3K79me3 only accumulated later in time (Fig. 4C). These observations are in agreement with the low *k_2_* of TbDot1A. Furthermore, the changing ratios between the individual H3K79 methylation states confirm the distributive nature of the enzyme. In the presence of TbDot1B, almost exclusively H3K79me3 was detected on new H3 (the H3K79me1 and H3K79me2 blots are essentially empty). Some H3K79me2 was detected early on (after one population doubling), while no H3K79me1 could be identified on old or new histone H3 (Fig. 4D). This is in agreement with the very high estimated values for *k_1_* and *k_2_* relative to that of *k_0_*. One consequence of the low *k_0_*, high *k_2_,* and very high *k_1_* of TbDot1B is that supply of H3K79me1 is slow while its conversion into H3K79me2 and further to H3K79me3 is rapid, leading to a seemingly binary switch from H3K79me0 to H3K79me3. As hDot1L had very low activity and methylated only a small percentage of histone H3 under steady-state conditions, we did not perform this assay for hDot1L. However, we conclude that the distributive mechanism and highly different catalytic activities predicted for yDot1, TbDot1A and TbDot1B based on steady state measurements are consistent with the kinetics of H3K79 methylation on newly synthesized histones.

### Simulations of H3K76 methylation in trypanosomes

**Figure 5:**
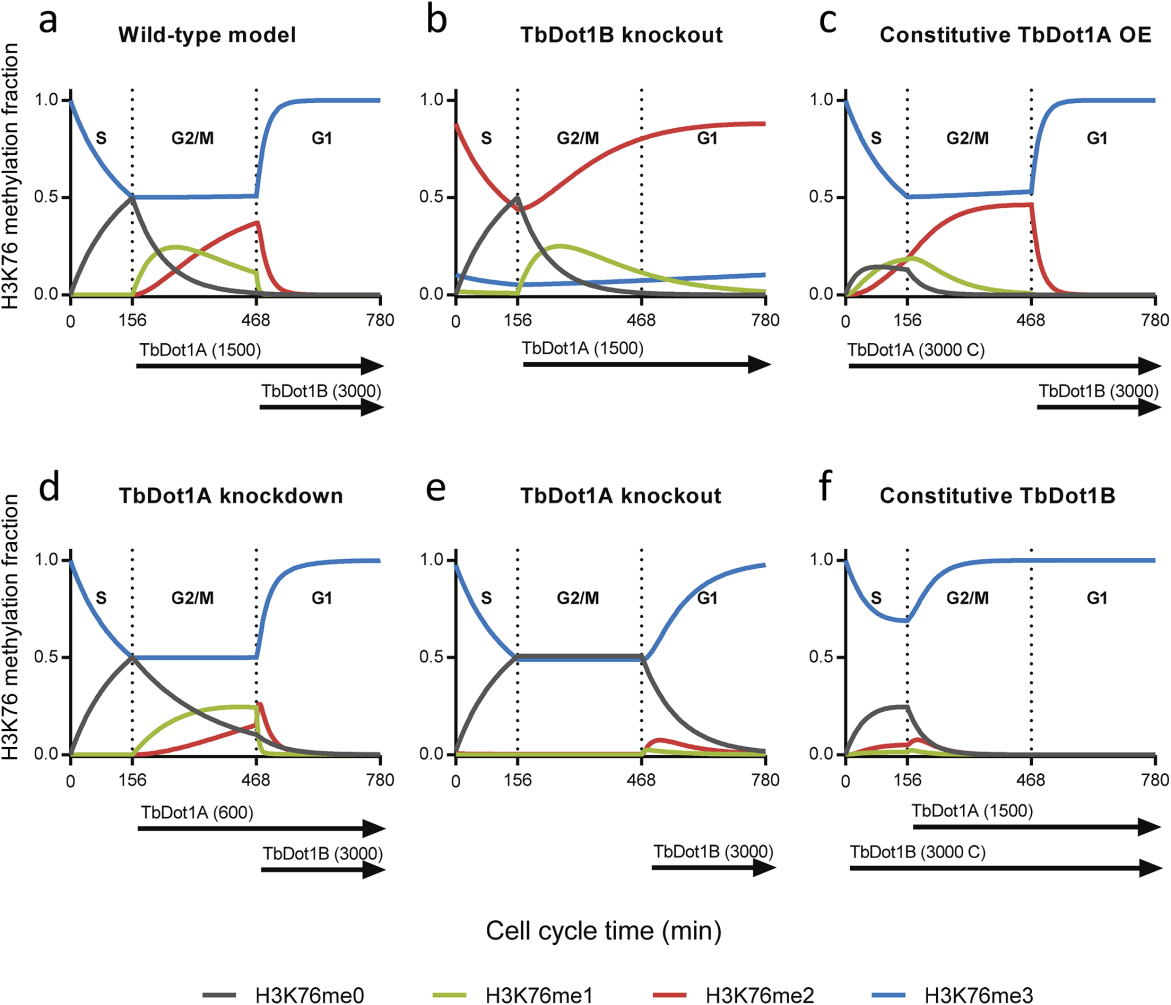
Simulation of H3K76 methylation throughout the trypanosome cell cycle. Simulation of H3K76 methylation in a 13-hour procyclic trypanosome cell cycle divided in an S-phase (20%), G2/M-phase (40%) and G1-phase (40%). TbDot1A and -B expression was manually set to approximately fit the reported experimental data^12^,^38^ (see main text). Furthermore, adjustments were made in the model to simulate H3K76 methylation patterns in TbDot1A or -B depleted or overexpressing trypanosomes. A) Expression of TbDot1A in G2/M and G1 with 1500 copies and expression of TbDot1B only during G1 with 3000 copies resulted in a model that simulated the experimental data best. B) TbDot1B was excluded from the model to simulate a TbDot1B knockout. C) To simulate TbDot1A overexpression studies, TbDot1A expression was increased to 3000 copies per cell and continuously expressed (c) during the cell cycle. D) To simulate TbDot1A knockdown studies, TbDot1A expression was lowered to 600 copies per cell. E) TbDot1A was excluded from the model to simulate a TbDot1A knockout. F) To simulate TbDot1B misregulation studies, TbDot1B was continuously expressed (c) during the cell cycle.

In trypanosomes, TbDot1-mediated H3K76 methylation is tightly linked to cell cycle progression^12^,^38^. To gain more insight into how the cell-cycle regulated pattern is generated and how sensitive it is to perturbation, the estimated rate constants were used to parameterize a model that represents H3K76 methylation in trypanosomes. The model accounts for one 13-hour cell cycle of procyclic (insect stage) trypanosomes^12^ in which the G2/M, S and G1 phases take up respectively 40%, 20% and 40% of the time^47^. Since no data were available for TbDot1 enzyme regulation or expression, TbDot1 cell cycle regulation and expression levels were adjusted in the model (Supplemental Fig. S6) until the simulations represented the H3K76 methylation states based on cell staining^12^,^38^ in trypanosomes (Fig. 5A and Supplemental Fig. S6; and see Supplemental Methods). For example, no H3K76me1 or -me2 has been observed in S phase, but H3K76me1 and -me2 accumulate during G2 followed by an H3K76me2 peak in mitosis^38^. To recapitulate this pattern in the model, TbDot1A becomes active after S-phase at the start of G2/M to generate H3K76me1 and H3K76me2 (Fig. 5A). Subsequently, H3K76me1 and -me2 signals rapidly decrease in G1. Therefore, in the model, TbDot1B becomes active at the start of G1 to mediate the rapid transition from H3K76me1 and -me2 to exclusively H3K76me3 during G1^12^ (Fig. 5A). The levels of TbDot1A and TbDot1B expression used in the model were validated by determining the consequences of moderate changes in the parameters. Changes in TbDot1A expression resulted in altered H3K76 methylation patterns that are in disagreement with experimental data (Fig. 5, Supplemental Fig. S6 and Supplemental Methods). The TbDot1B copy number in the model was less critical than that of TbDot1A because a small increase or decrease in TbDot1B levels did not affect the efficient transition to H3K76me3. Also, alternative timing of TbDot1A and -B during the cell cycle resulted in models that did not recapitulate the observed H3K76 methylation pattern in wild-type trypanosomes (Supplemental Fig. S6). Although attempts to detect endogenous TbDot1A/B expression as a function of the cell cycle in trypanosomes have failed^12^, it will be interesting to obtain more information on the regulation of the enzymes during the cell cycle in future studies.

Next, we examined whether parameter variations that mimic changes in TbDot1A or -B expression in trypanosomes recapitulate the altered H3K76 methylation signals observed in trypanosomes. First, deletion of TbDot1B from trypanosomes results in loss of H3K76me3 and enhanced H3K76me2 signals^12^. This is adequately recapitulated by our model in which H3K76me2 accumulates during G1 in the absence of H3K76me3 (Fig. 5B). Second, trypanosome cells constitutively overexpressing TbDot1A have enhanced H3K76me2 signals in S- and G2-phases^38^. Similarly, our model predicts enhanced H3K76me2 (and -me1) during S-phase and G2/M upon TbDot1A overexpression (Fig. 5C). We conclude that our model predicts H3K76 methylation states in trypanosomes accurately. Furthermore, the model indicates that TbDot1A functions as a timer in G2/M through the consecutive establishment of an H3K76me1 peak during G2 and an H3K76me2 peak during mitosis (Fig. 5A).

Finally, we used the model to gain insight into several experimental TbDot1A/B perturbations that cause trypanosome lethality or sickness. First, depletion of TbDot1A causes replication problems^38^. Our model for procyclic cells predicts a reduction in H3K76me2 levels upon a 2.5-fold reduction of TbDot1A (Fig. 5D). In addition to this expected abundance change^12^,^38^, our model also shows altered timing of the H3K76me1 and –me2 signals. Second, ectopic expression of TbDot1A causes constitutive replication^38^. In addition to the expected increase in overall levels of H3K76me2 (area under the red curve in Fig. 5C) there is a predicted premature appearance of H3K76me2 in S-phase. Interestingly, the computational model also suggests that one of the predominant changes under those conditions is the premature appearance of H3K76me1 in S-phase (Fig. 5C). Since a premature predicted appearance of H3K76me2 in S-phase in TbDot1B knock-out cells is not associated with replication problems (Fig. 5B), this suggests that H3K76me1 and not H3K76me2 might be the signal causing replication defects in cells with ectopic TbDot1A expression. Third, knock-out of TbDot1A or constitutive overexpression of Dot1B is lethal^12^,^38^. According to the model, deletion of TbDot1A leads to loss of H3K76me2, whereas constitutive TbDot1B expression leads to increased H3K76me3. However, the model suggests that also the H3K76me1 levels during the cell cycle are severely affected (Fig. 5E-F) under both conditions. In summary, TbDot1A/B perturbations that according to the model change H3K79me1 timing in S-phase or G2/M are lethal (TbDot1A knock out, ectopically expressed TbDot1A, or ectopically expressed TbDot1B) (Fig. 5). The presence of H3K76me1 in S-phase upon constitutive TbDot1A expression might cause continuous replication and thereby lethality^12^,^38^. Similarly, the delay in the H3K76me1 signal in a TbDot1A-depleted cell might be responsible for the abolished replication^12^,^38^.

### Effect of excess H3K79me2 and H3K79me3 on yeast growth and silencing

**Figure 6:**
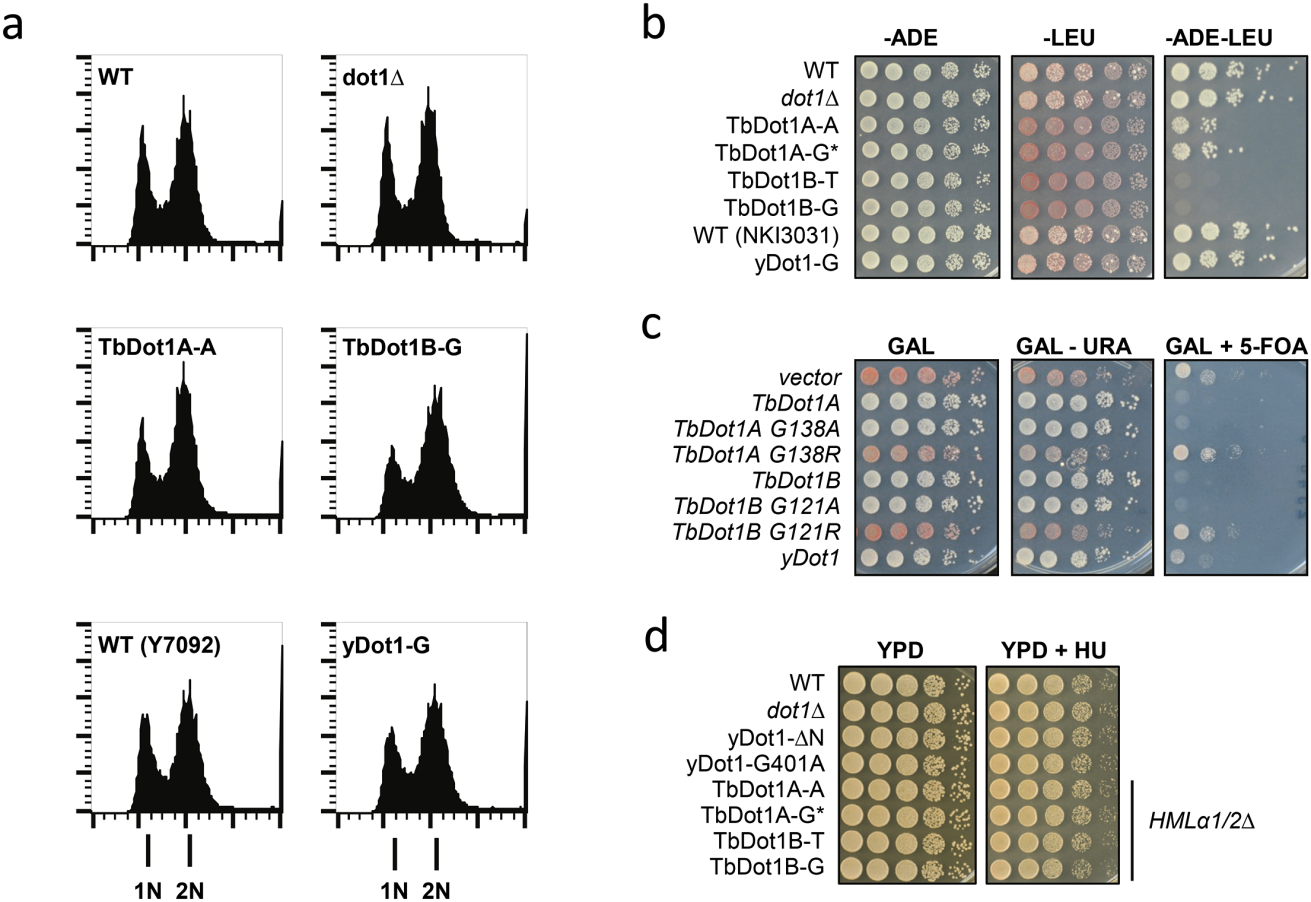
Expression of TbDot1A or TbDot1B in yeast affects silencing but not replication. A) Cell cycle progression of yeast strains overexpressing yDot1, TbDot1A or TbDot1B was determined by flow cytometry (FACS) analysis of DNA content and compared to the wild-type strain NKI6061 (WT). X-axis depicts DNA signal, Y-axis depicts cell count. The promoter from which the Dot1 enzymes were expressed is indicated behind the dash: A = ADHpr, G = GPDpr, D = DOT1pr. Strain Y7092 was used as the WT control for yDot1-G. B) To test whether the altered H3K79 methylation profiles affected silencing of the silent *HML*α mating locus yeast strains expressing yDot1, TbDot1A or TbDot1B in a *MAT*a *ADE2 + leu2Δ* background were mated with a WT strain with a *MAT*α *ade2Δ LEU2 +* background (BY4726). Loss of *HML*α silencing in *MAT*a cells leads to loss of mating type identity and loss of mating ability. Cells were plated in 10-fold serial dilutions on selective synthetic media. Diploids that result from effective mating were *ADE2 + LEU2 +* and able to grow on –ADE –LEU media. C) Telomeric silencing was examined in a strain (UCC7164) containing a telomeric URA3 reporter (TEL-VII-L) and telomeric *ADE2* reporter (ADE2-TEL-VR). Plasmid-based Dot1 expression was induced with 3% galactose^53^. Cells were plated in 10-fold serial dilutions on selective synthetic media with or without 5-FOA. Cells that silence *URA3* can grow on 5-FOA media whereas cells that express *URA3* cannot. Cells that silence ADE2 accumulate a red pigment whereas cells that express *ADE2* are white. TbDot1A G138A and TbDot1B G121A show H3K79 methylation activity, while TbDot1A 138R and TbDot1B G121R have lost their ability to methylate H3K79^53^. D) Yeast strains expressing yDot1, no Dot1, partially active yDot1 (ΔN and G401A)^21^, TbDot1A or TbDot1B were plated in 10-fold dilution series on rich media with or without 100 mM hydroxyurea (HU). Loss of mating type identity by TbDot1A or –B expression (see panel B) was eliminated by deletion of the *HML*α1/2 genes.

Having created yeast strains with unusually high H3K79 methylation levels, and given the proposed role of yDot1 in yeast cell cycle progression and gene silencing^2^,^25^,^48^,^49^,^50^,^51^,^52^, we examined whether conditions of excess H3K79me2 and H3K79me3 affected growth or gene silencing. In yeast cells expressing TbDot1A, the H3K79me2 levels are ~80%, which is ~4 fold higher than wild type. In yeast cells expressing TbDot1B, virtually all H3 is trimethylated, which is ~2 fold higher than wild-type cells (e.g. see Supplemental Table S1). Remarkably, in these cells no cell-cycle changes were observed by FACS analysis of DNA content (Fig. 6A). Also in limiting dilution spot tests no growth defects were found in the absence or presence of DNA replication inhibitor hydroxyurea. (Fig. 6B–D). These results suggest that in budding yeast hyper H3K79 methylation is not detrimental for cell-cycle progression. However, silencing of telomeric reporter genes and the native mating type locus *HMLα* was severely compromised by TbDot1A or –B expression (Fig. 6B–C). These results suggest that H3K79me3 as well as H3K79me2 can act as disruptors of silencing. The strong desilencing effects also suggest that TbDot1A and TbDot1B are efficient competitors of the SIR complex, which binds to and silences telomeric regions and mating type loci. Yeast Dot1 overexpression affected silencing much less, even though this also led to a global increase in H3K79me3. In contrast to yDot1, the trypanosome Dot1 proteins do not require the N-terminal tail of histone H4^53^, a part of the nucleosome important for recruitment of the SIR complex. Therefore, it is possible that TbDot1A and TbDot1B, in contrast to yDot1, are able to methylate regions occupied by the SIR complex and thereby weaken the silencing activity of the SIR complex.

## Discussion

The mechanism of action of histone methyltransferases has been subject of intensive research^3^,^20^,^21^,^22^,^23^,^24^,^25^,^26^,^27^,^28^,^29^,^30^,^31^,^32^. Here, we report that Dot1 enzymes from yeast, trypanosomes and humans share a distributive mechanism. However, the four Dot1 enzymes with a conserved catalytic domain generate highly divergent H3K79 methylation patterns upon expression in yeast, which is a consequence of the specific activities of each enzyme.

Our findings that TbDot1 enzymes function in a distributive manner in living cells are in agreement with very recent studies showing that both TbDot1 enzymes methylate recombinant trypanosome nucleosomes *in vitro* in a distributive manner^23^. In our *in vivo* experiments we obtained data that enabled the determination of estimated rate constants for TbDot1A and TbDot1B. This yielded several new important insights. First, TbDot1A was less efficient than TbDot1B and yDot1 in generating H3K79me3. However, it generated substantial amounts of H3K79me3 upon increasing concentrations. In contrast, in the same cellular environment, hDot1L was unable to generate H3K79me3. This was unexpected because hDot1L, like TbDot1B, contains a conserved tyrosine in the D-loop of its methyltransferase domain that was recently suggested to be important for trimethylase activity^23^ whereas TbDot1A lacks this residue. Second, TbDot1B is less efficient in generating H3K79me1 but rapidly generates H3K79me3. The structural basis for the different substrate specificities of TbDot1A and TbDot1B was recently demonstrated by elegant homology-based modeling experiments and *in vitro* assays^23^. Interestingly, our experiments show that TbDot1B also generates H3K79me2 extremely efficiently (Table I). In accordance with our predictions, converting TbDot1A into a better trimethylase by domain swaps with TbDot1B also transformed TbDot1A into an enzyme with higher dimethylation rates *in vitro*^23^. A high dimethylase activity of TbDot1B may be unexpected since TbDot1A was shown to be the main generator of H3K76me2 in trypanosomes^12^. In view of the distributive mechanism of TbDot1A and TbDot1B, it is logical, however, that TbDot1B requires efficient H3K76me2 formation. TbDot1B needs to remove any remaining H3K76me1 that was not yet converted to H3K76me2 by TbDot1A to obtain an H3K79me2 substrate for further conversion to the final trimethylation state (see Fig. 5).

The insights into the catalytic activity of the TbDot1 enzymes in combination with the simulations of H3K76 methylation throughout the trypanosome cell cycle has revealed several interesting features about the dynamics and regulation of H3K76 methylation. First, TbDot1A seems to function as a molecular timer like yDot1^21^ and efficiently generates two distinct methylation states during G2/M (first H3K76me1, then H3K76me2) without the need for additional factors that regulate H3K76me levels, such as putative demethylases. The predicted temporal control of H3K76me1 during the *T. brucei* cell cycle and the association with replication errors and viability (see Results section above) suggests that it may serve as a specific critical signal. In this view, the high rate constant of TbDot1B for the synthesis of H3K79me2 (*k_1_*) might be required to rapidly remove any remaining H3K76me1 at the onset of G1.

A second interesting feature is that co-operation of two distributive Dot1 enzymes is needed to generate a cell cycle dependent H3K76 methylation pattern. This implies that the system is flexible but also sensitive to perturbations and might require additional stabilizing mechanisms. For example, a prolonged cell cycle affects the H3K79 methylation pattern in yeast^21^ and could possibly affect H3K76me1 and -me2 in trypanosomes during G2/M as well. In contrast, the global H3K76me3 signal during the cell cycle seems less sensitive to perturbations as moderate changes in expression of TbDot1A or TbDot1B, or the loss of TbDot1A, have minor effects on the rapid global trimethylation of H3K76 (Fig. 5 and Supplemental Fig. S6). Thus, the model suggests that the timing of both TbDot1A and -B and the expression level of TbDot1A is tightly regulated during the cell cycle (Supplemental Fig. S6).

Finally, lowering the average expression but not changing the timing of TbDot1A and -B activity in the model progressively disturbs the cell cycle regulated H3K76 methylation states but not the global methylation levels (Supplemental Fig. S7). This indicates that global H3K76 methylation levels may be a sub-optimal reporter of Dot1 inhibition in Trypanosomes. The data we presented here start to unravel the importance of H3K76 methylation and TbDot1A and -B regulation in trypanosomes. Further *in vivo* experiments will be important for validation of our predictions.

Our findings suggest that hDot1L also acts by a distributive mechanism. This is in agreement with recent biochemical and structural studies showing that hDot1L has an occluded active site. Most likely, hDot1L requires conformational rearrangements to allow for binding of the substrate lysine and release of the reaction by-product S-adenosyl-homocysteine (SAH) and reloading of the methyl donor S-adenosyl-methionine (SAM)^18^,^24^. The catalytic domain of hDot1L expressed in yeast has relatively low activity when compared to the other Dot1 enzymes investigated here. However, the observed abundance of lower H3K79 methylation states is generally in line with the steady-state H3K79 methylation patterns observed in human cells^37^. For example, HeLa cells have a H3K79 methylation pattern that consists of 77% unmethylated H3, 20% H3K79me1, and 2.5% H3K79me2, whereas H3K79me3 was not detected by mass spectrometry^37^. In its native environment in the cell, hDot1L activity may be influenced by its assembly into one of the complexes in which it has been found^7^,^54^,^55^,^56^. hDot1L has been shown to be assembled into the Dot1.Com complex and interact with RNA polymerase II, AF9, AF10, and Bat3^8^,^57^,^58^,^59^. Such interactions might facilitate the conformational changes in the hDot1L structure needed to allow for binding and releasing SAM and SAH, respectively^18^,^24^. Protein-protein interactions also target hDot1L to specific sites in the genome^8^,^57^,^58^,^59^, thereby locally increasing H3K79 methylation. It remains to be investigated if hDot1L in such a complex is still distributive or constitutes a pseudo-processive mechanism. Indeed, hDot1L and RNA polymerase II colocalize at transcription start sites, where H3K79me2 is enriched^6^,^8^,^58^,^60^ and recruitment of hDot1L to H3K9-acetylated chromatin through AF9 correlates with the presence of H3K79me3^59^. Therefore, it is possible that within the cell a certain fraction of the hDot1L enzyme pool acts in a pseudo-processive manner and that similar rules apply to Dot1 proteins in other species.

Our analysis of Dot1 proteins with highly distinct catalytic properties from evolutionary distinct organisms shows that Dot1 enzymes across species share a distributive mode of action. As has been the case for yeast Dot1, the insights in the catalytic mechanism of Dot1 enzymes in humans and trypanosomes will help to further unravel and understand the mechanisms that regulate the activity of H3K79/76 methylation in health and disease.

## Supplementary Material

Supplementary Information Supplementary Information

## Figures and Tables

**Table 1:** Predicted rate constants (µM^–1^min^–1^) for Dot1 enzymes

H3K79me	k_x_	yDot1*	TbDot1A	TbDot1B
**H3K79me0**				
↓	**k_0_**	0.050 (0.055)	0.045	0.018
**H3K79me1**				
↓	**k_1_**	0.021 (0.019)	0.022	0.32
**H3K79me2**				
↓	**k_2_**	0.010 (0.0087)	0.00037	0.078
**H3K79me3**				

*values indicated between brackets were reported in De Vos et al, 2011.
