# Supplementary material for: Dot1 histone methyltransferases share a distributive mechanism but have highly diverged catalytic properties
Source: Sci Rep. 2015 May 12;5:9824. doi: 10.1038/srep09824 (PMC4650758; doi:10.1038/srep09824)
Supplement: Supplementary Information [file srep09824-s1.pdf]

Supplemental Information

Dot1 histone methyltransferases share a distributive mechanism but have highly diverged catalytic properties.

Iris J.E. Stulemeijer[1*], Dirk De Vos[2], Kirsten van Harten[1], Onkar K. Joshi[1], Olga Blomberg[1], Tibor van Welsem[1], Marit Terweij[1], Hanneke Vlaming[1], Erik L. de Graaf[3], A.F. Maarten Altelaar[3], Barbara M. Bakker[4], Fred van Leeuwen[1*]

Supplemental Figure S1

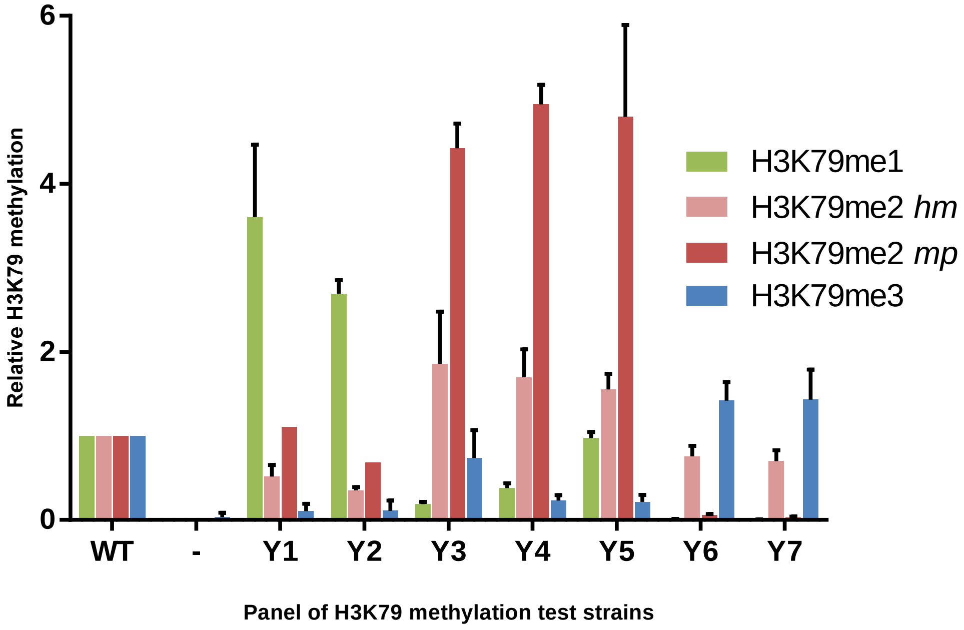

Supplemental Figure S2

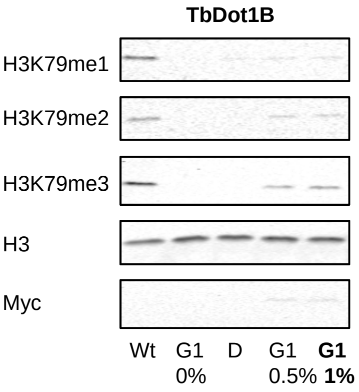

Supplemental Figure S3

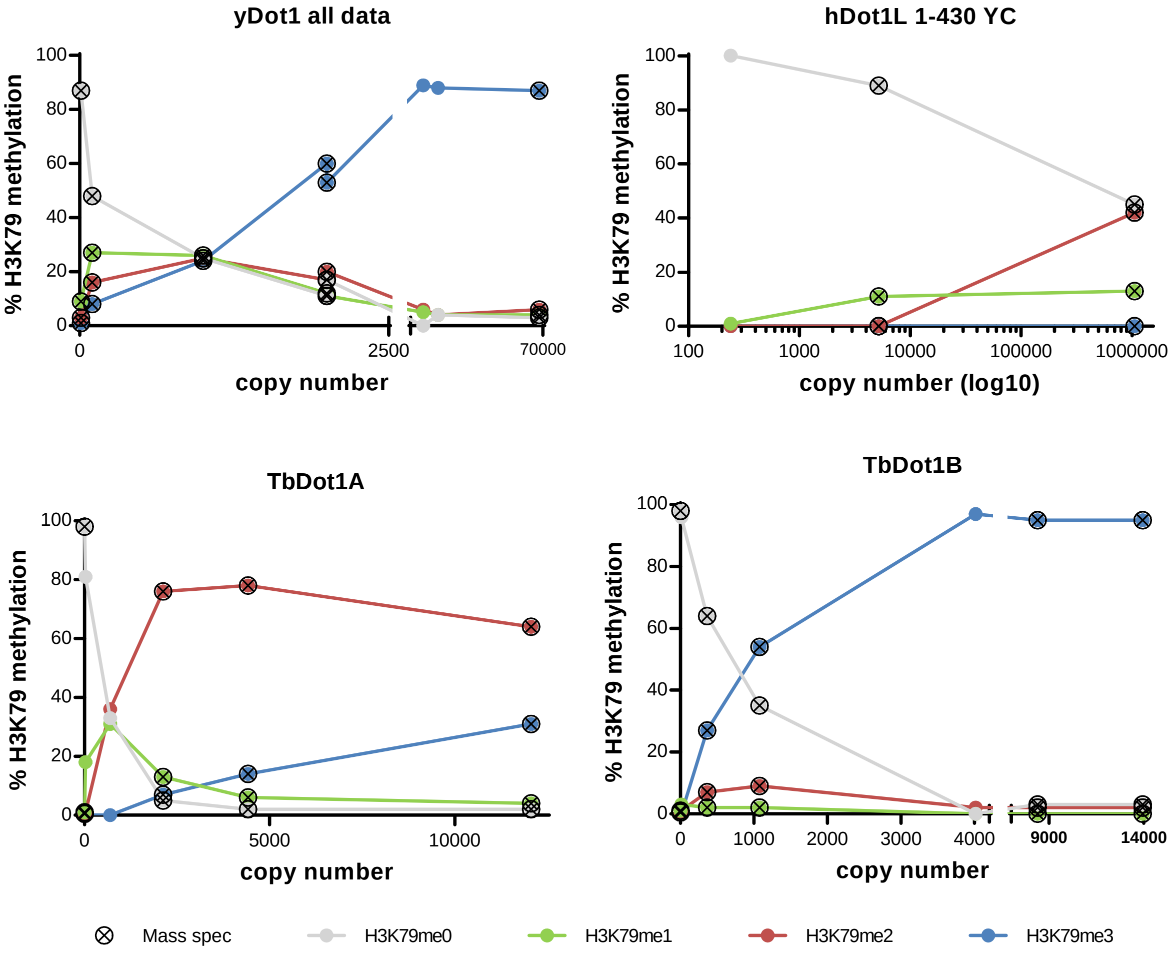

Supplemental Figure S4

a

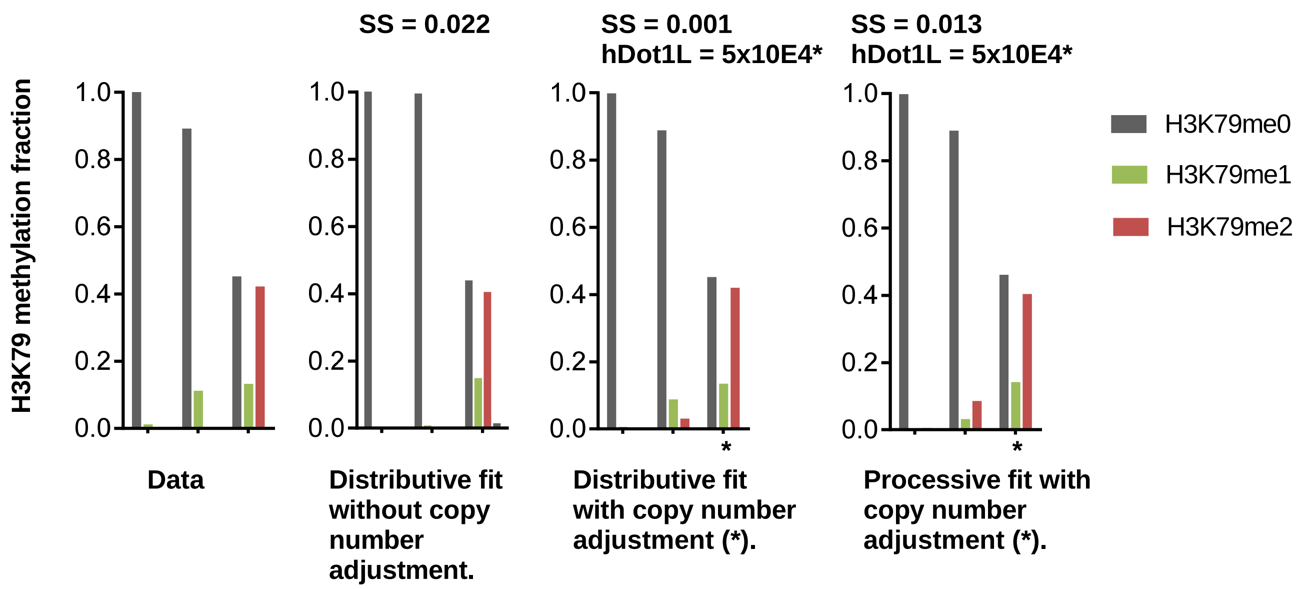

b

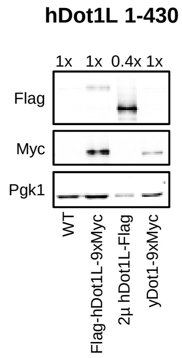

Supplemental Figure S5

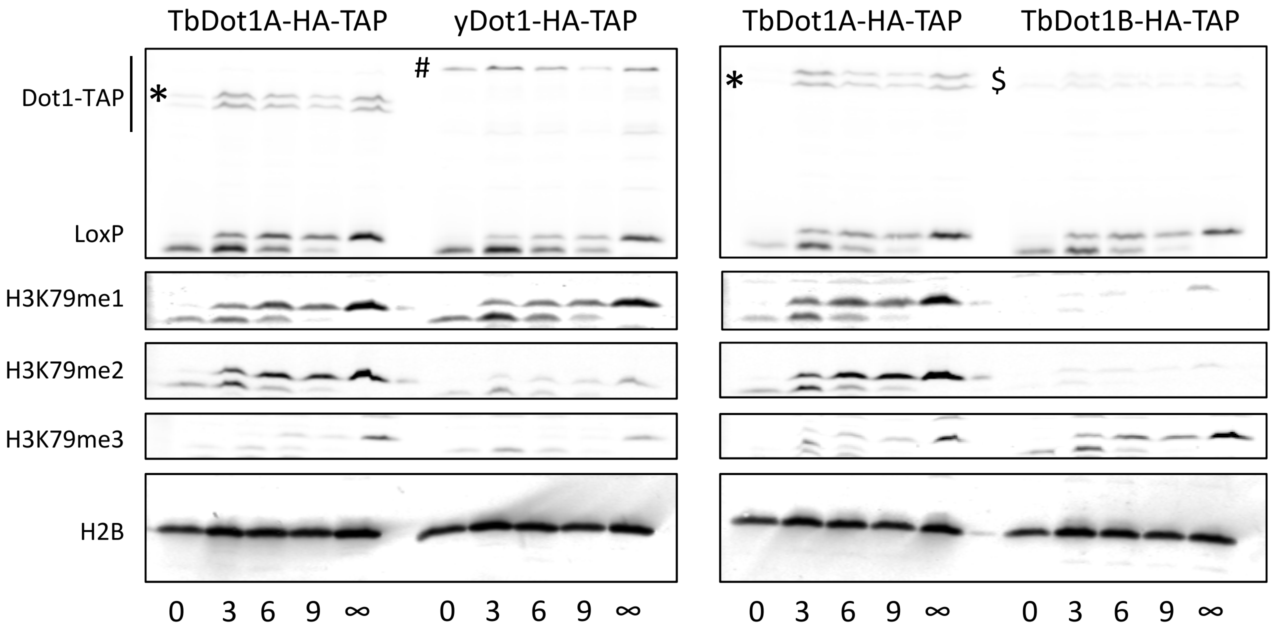

Supplemental Figure S6

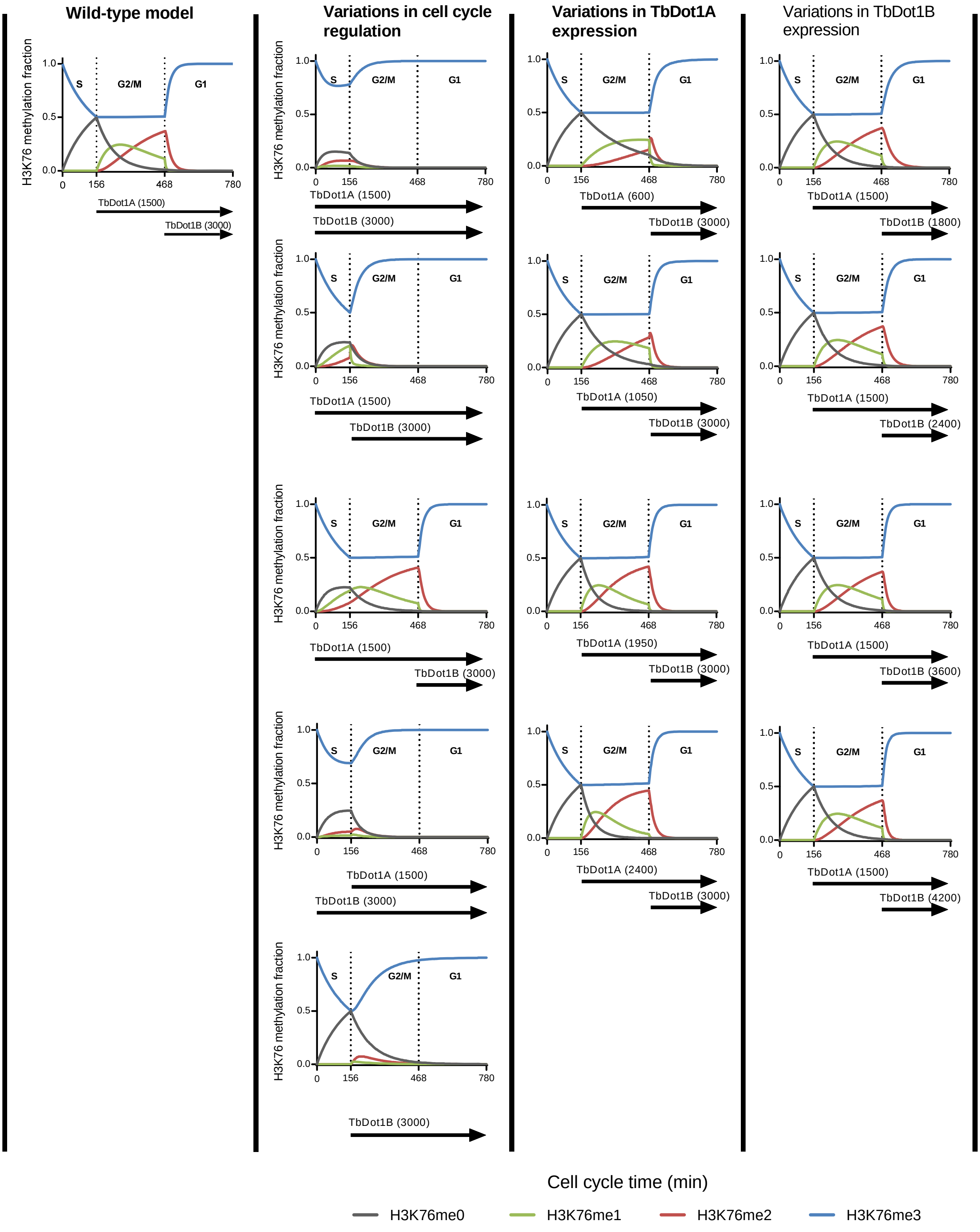

Supplemental Figure S7

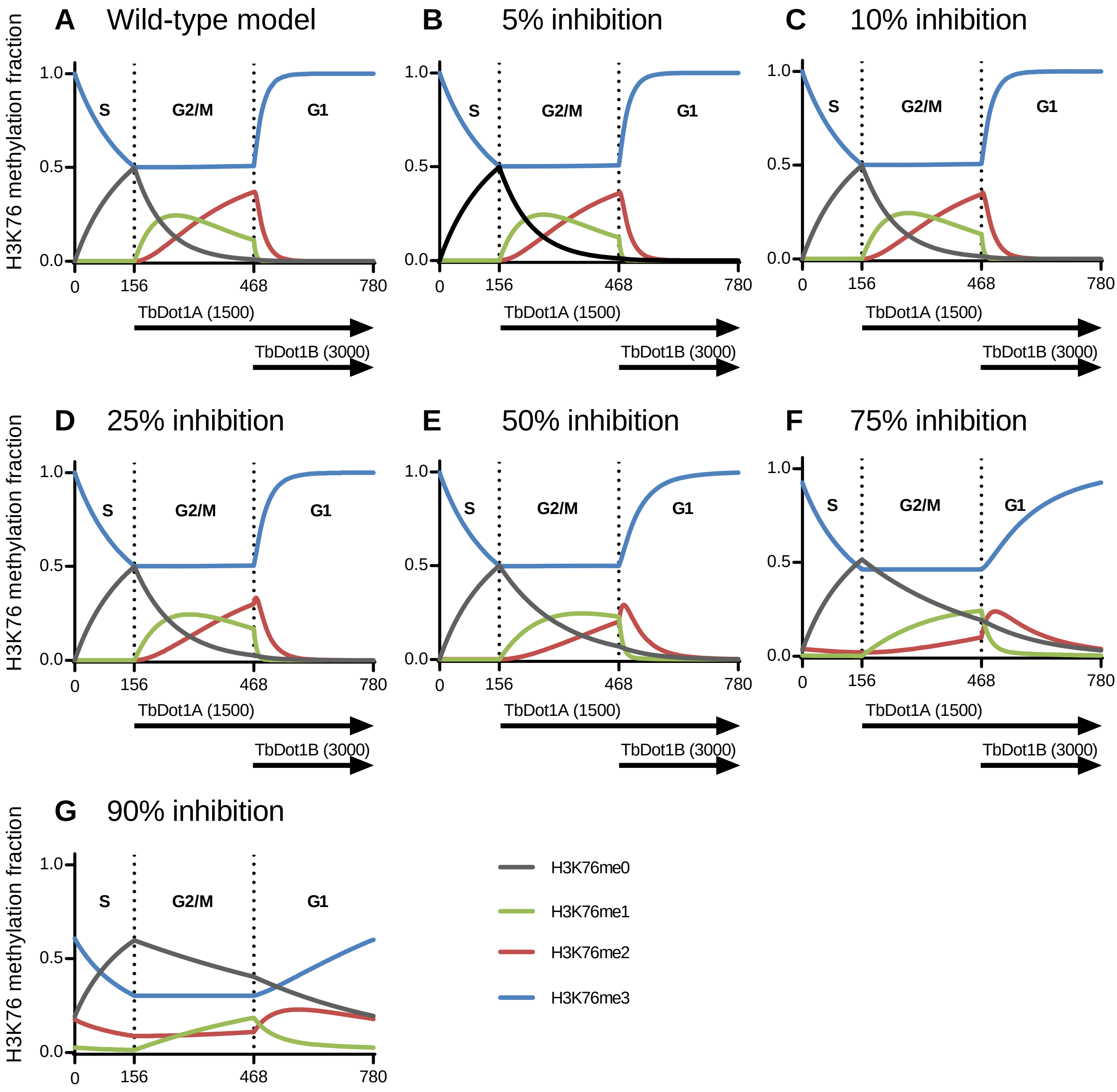

Supplemental Figure legends

Figure S1. Linearity of H3K79 methylation antibodies.
Quantified western-blot data using an Odyssey scanner of at least two replicates of the yeast-strain series shown in Figure 1C to determine the linearity of the H3K79me1, -me2 and –me3 home-made antibodies [1] and the H3K79me2 antibody from Millipore.

Figure S2. H3K79 methylation under conditions of low TbDot1B expression.
Western-blot analysis of H3K79 methylation states of TbDot1B expressed from a galactose-inducible *GAL1* promoter (G1) or the *yDOT1* promoter (D) at the *yDOT1* locus in *S. cerevisiae.* TbDot1B copy-number expression was determined by detection of the c-terminal 9xMyc tag and comparison to yDot1-9xMyc expression (not shown). H3 and Pgk1 were used as loading controls. H3K79 methylation states were compared to a wild-type yeast strain (WT), and a non-induced stain (G1, 0%) was included to determine antibody specificity. TbDot1B expression from the *GAL1*pr using 1% galactose (and 1% glucose) in the medium resulted in an H3K79 methylation pattern in between that of the H3K79 methylation patterns generated when TbDot1B was expressed from the *DOT1*pr (D) or the *TEF*pr + *yDOT1* 5'UTR (T*). H3K79 methylation patterns of TbDot1B induced by 1% galactose are shown in Figure S3 as the second data points on the x-axis.

Figure S3. Quantified H3K79 methylation patterns of yeast strains expressing yDot1, TbDot1A, TbDot1B and hDot1L.
H3K79 methylation patterns shown in Figures 1, 2, and S2 were quantified using the validated antibodies described in Figure 1C and 1D or measured by MS. A data quality check was performed as described in the methods and non-reproducible H3K79 methylation patterns were removed from the dataset. To visualize the correlation between Dot1 expression and H3K79 methylation patterns, the H3K79 methylation states were plotted against the Dot1 expression. Data points indicated by ⊗ represent H3K79 methylation states measured by mass spectrometry.

Figure S4. Simulations for hDot1L
A) Simulations of the H3K79 methylation pattern with *dot1Δ*, intermediate (single integration) hDot1L expression, and high hDot1L expression (2µ plasmid) in minimal medium using 2% galactose. Simulations (second panel) could not fit the experimental data (first panel) properly. Likely, the estimated copy number is not representative for the active hDot1L fraction in the cell since the estimated enzyme concentration is ~5 fold higher than the substrate (H3) concentration. Fortunately, the H3K79 methylation patterns contained sufficient information to simultaneously fit the methylation rate constants for hDot1L and the hDot1L copy number of the multi-copy plasmid with the integrated hDot1L copy number as a reference. In the absence of any observed H3K79me3, the k_2 parameter was set to zero for numeric stability. This approach resulted in an improved fit to the experimental data (third panel) and yielded an estimate of the (effective) hDot1L copy number in the multi-copy plasmid expressing strain of about 20 fold lower than the estimate based on the western blot. The distributive model performed better than a processive model with the same adjustments (fourth panel).

B) hDot1L was expressed in yeast from the *GAL1* promoter at the endogenous *yDOT1* locus (Flag-hDot1L-9xMyc) or at a multi-copy plasmid (2µ-hDot1L-Flag) upon induction by 2% galactose as the sole carbon source in the minimal medium. H3K79me levels are shown in Fig. 2D. *Samples from 2µ-hDot1L-Flag were diluted 2.5-fold for Flag, Myc and Pgk1 detection to visualize the extreme overexpression of hDot1L. For copy number estimates, yDot1-9xMyc (2000 copies/cell) was used as a reference for Flag-hDot1L-9xMyc (Myc tag); 2µ-hDot1L-Flag was compared to Flag-hDot1L-9xMyc (Flag tag).

Figure S5. Relative H3K79 methylation signals on new histones.
Western-blot analysis of H3K79 methylation patterns on old (upper band) and new (lower band) histone H3 by the indicated Dot1 enzymes. The left TbDot1A panel and the TbDot1B panel are identical to the panels shown in Figure 4C and 4D, respectively. Dot1 enzyme expression was constitutive during the experiment, which was confirmed by IgG-mediated recognition of the TAP tag fused to the Dot1 enzymes. yDot1 (#), TbDot1A (*) and TbDot1B ($) signals are indicated on the blot. Note that yDot1 has reduced activity upon tagging, which results in enhanced H3K79me1 and decreased H3K79me3 signals. Samples were taken upon one, two or three doublings of the population of cells (Doubling = 1, 2, or 3). As controls, samples were taken immediately following the addition of β-estradiol (100% "old", Doubling = 0), and from a strain that expressed only the H3-HAHis (100% "new", ∞). H2B was used as loading control.

Figure S6. Establishment of a model for H3K76 methylation during the trypanosome cell cycle.
A model of the trypanosome cell cycle was established. The model accounts for one 13-hour cell cycle of procyclic cells [2] in which the G2/M, S and G1 phases take up respectively 40%, 20% and 40% of the time [3]. To simulate H3K76 methylation such that it represents H3K76 methylation states based on cell staining [2,4] in trypanosomes (see Supplemental Methods for H3K76 methylation state observations and model assumptions), TbDot1 cell cycle regulation and expression levels were varied in the model. The conditions shown in the wild-type model (box; also shown in Figure 5A) represent the observed H3K76 methylation patterns in trypanosomes best. Variations in the timing of TbDot1A and –B expression (Variations in cell cycle regulation) do not match the observed trypanosome H3K76 methylation pattern as H3K76me1 and –me2 occurred during S-phase and/or H3K76me2 is absent during mitosis. Variations in the expression level of TbDot1A and –B are also addressed. TbDot1A expression levels lower than in the wild-type model result in a peak of H3K76me2 at the start of G1 while higher expression levels enhance H3K76me2 levels during G2. *In vivo*, H3K76me2 is mainly detected during mitosis (see Supplemental methods). Variations in TbDot1B expression level in G1 only have a small effect in the model on the rate at which all histones are fully converted to H3K76me3. Therefore, the expression level of TbDot1B is probably less tightly regulated than the expression level of TbDot1A.

Figure S7. Inhibition of both TbDot1A and –B to determine robustness of the H3K76 methylation pattern. The trypanosome cell cycle model for H3K76 methylation (Figure 5A) was adjusted to simulate inhibition of both TbDot1A and TbDot1B simultaneously, mimicking the addition of a putative trypanosome Dot1 inhibitor. TbDot1A and –B activity was reduced by

lowering the copy number in the model by 0% (A; Wild-type model), 5% (B), 10% (C), 25% (D), 50% (E), 75% (F) and 90% (G).

Supplemental Table SI

Mass spectrometry

Strain	Genotype	Average % H3K79me0	H3K79me1	H3K79me2	H3K79me3	SEM H3K79me0	H3K79me1	H3K79me2	H3K79me3
NKI6061	Wild-type	17	11	20	53	0.71	0.00	0.00	1.41
NKI6069	*dot1* KO	98	1	1	1	0.71	0.00	0.71	0.71
NKI6081	*DOT1* pr-Dot1-172-582	38	43	19	2	4.95	3.54	0.71	0.00
NKI6077	*DOT1* pr-Dot1-G401A	53	32	14	2	2.83	1.41	0.71	0.00
NKI6084	*TEF* pr-TbDot1A	2	4	64	31	0.71	0.71	0.00	0.00
NKI6099	*ADH* pr-TbDot1A	2	6	78	14	0.00	0.00	1.41	2.12
NKI6100	*GPD* pr 5'UTR-TbDot1A	5	13	76	7	1.41	0.71	2.83	1.41
NKI6085	*TEF* pr-TbDot1B	3	0	2	95	0.00	0.00	0.00	0.00
NKI6083	*GPD* pr-TbDot1B	3	0	2	95	0.00	0.00	0.00	0.00
NKI8046	*GPD* pr-yDot1	5	4	6	85	0.00	0.00	0.00	0.00

Table SII_Plasmids and Strains

Plasmid	Description	Reference
pYM-N6	KanMX4 *ADH* -promoter	Janke et al, 2004; Yeast
pYM-N11	natNT2 *CYC1* -promoter	Janke et al, 2004; Yeast
pYM-N14	KanMX4 *GPD* -promoter	Janke et al, 2004; Yeast
pYM-N18	KanMX4 *TEF* -promoter	Janke et al, 2004; Yeast
pYM20	hphNT1 9Myc tag	Janke et al, 2004; Yeast
pTW103	URA3 *GPD* -promoter	this study
pIS013	2µ TRP1 *GAL1* pr-NLS-3xFLAG-*CYC1* term	this study
pTW125	2µ TRP1 hDot1L 1-430-NLS-3xFLAG	this study
pFvl925	2µ-LEU2-*ADH* pr-LexA-V5-*hDot1L1-430*	Stulemeijer et al, 2011; Epigenetics and Chromatin
pFvl261	CEN-*TRP1* -*GAL1* pr	Frederiks et al, 2010; Journal of Cell Science
pCJ49	CEN-*TRP1* -*GAL1* pr-*TbDot1A* -HA	Frederiks et al, 2010; Journal of Cell Science
pCJ50	CEN-*TRP1* -*GAL1* pr-*TbDot1A G138A* -HA	this study
pFF020	CEN-*TRP1* -*GAL1* pr-*TbDot1A G138R* -HA	Frederiks et al, 2010; Journal of Cell Science
pTW100	CEN-*TRP1* -*GAL1* pr-*TbDot1A* -HA-TAP	Frederiks et al, 2010; Journal of Cell Science
pFF019	CEN-*TRP1* -*GAL1* pr-*TbDot1B* -HA	Frederiks et al, 2010; Journal of Cell Science
pCJ52	CEN-*TRP1* -*GAL1* pr-*TbDot1B G121A* -HA	this study
pFF021	CEN-*TRP1* -*GAL1* pr-*TbDot1B G12RA* -HA	Frederiks et al, 2010; Journal of Cell Science
pTW098	CEN-*TRP1* -*GAL1* pr-*TbDot1B* -HA-TAP	Van Leeuwen et al, 2002; Cell
pFF018	2µ *TRP1-GAL1* pr-*yDot1*	Frederiks et al, 2010; Journal of Cell Science
pRS306	2µ-*URA3*	Sikorski et al, 1989; Genetics
pRS400	2µ-KanMX	Sikorski et al, 1989; Genetics
pUC57-NLSTAG3e	spacer-NLS-3xFLAG	this study
pTCG	2µ-*TRP1-GAL1* pr-MCS-*CYC1* term	Peterson et al, 2001; Nature Genetics
pcDNA3b-FLAG-hDot1L	Flag-hDot1L	Feng et al, 2002; Current Biology
pFF008	pET16b-*hDOT1L 1-466*	this study

Strain	Genotype	Reference
NKI6061	MATa his3-1 leu2-0 lys2-0 met15d0 trp1d63 ura3-0 arg4::KanMX	this study
NKI6151	MATa his3-1 leu2-0 lys2-0 met15d0 trp1d63 ura3-0 arg4::KanMX URA3-GPDpr-Dot1	this study
NKI6069	MATa his3-1 leu2-0 lys2-0 met15d0 trp1d63 ura3-0 arg4::KanMX dot1::URA3	this study
NKI6077	MATa his3-1 leu2-0 lys2-0 met15d0 trp1d63 ura3-0 arg4::KanMX dot1::Dot1-G401A	this study
NKI6081	MATa his3-1 leu2-0 lys2-0 met15d0 trp1d63 ura3-0 arg4::KanMX dot1::Dot1-172-582	this study
NKI6078	MATa his3-1 leu2-0 lys2-0 met15d0 trp1d63 ura3-0 arg4::KanMX dot1::TbDot1A	this study
NKI6082	MATa his3-1 leu2-0 lys2-0 met15d0 trp1d63 ura3-0 arg4::KanMX dot1::NatMX-GPDpr-TbDot1A	this study
NKI6084	MATa his3-1 leu2-0 lys2-0 met15d0 trp1d63 ura3-0 arg4::KanMX dot1::NatMX-TEFpr-TbDot1A	this study
NKI6099	MATa his3-1 leu2-0 lys2-0 met15d0 trp1d63 ura3-0 arg4::KanMX dot1::NatMX-ADHpr-TbDot1A	this study
NKI6114	MATa his3-1 leu2-0 lys2-0 met15d0 trp1d63 ura3-0 arg4::KanMX dot1::NatMX-ADHpr-TbDot1A HMLα1/2Δ::HphMX	this study
NKI6100	MATa his3-1 leu2-0 lys2-0 met15d0 trp1d63 ura3-0 arg4::KanMX dot1::NatMX-GPDpr-5' UTR-TbDot1A	this study
NKI6115	MATa his3-1 leu2-0 lys2-0 met15d0 trp1d63 ura3-0 arg4::KanMX dot1::NatMX-GPDpr-5' UTR-TbDot1A HMLα1/2Δ::HphMX	this study
NKI6101	MATa his3-1 leu2-0 lys2-0 met15d0 trp1d63 ura3-0 arg4::KanMX dot1::NatMX-TEFpr-5' UTR-TbDot1A	this study
NKI6145	MATa his3-1 leu2-0 lys2-0 met15d0 trp1d63 ura3-0 arg4::KanMX dot1::TbDot1A-9xmyc-HphNt1	this study
NKI6146	MATa his3-1 leu2-0 lys2-0 met15d0 trp1d63 ura3-0 arg4::KanMX dot1::NatMX-TEFpr-5' UTR-TbDot1A-9xmyc-HphNt1	this study
NKI6147	MATa his3-1 leu2-0 lys2-0 met15d0 trp1d63 ura3-0 arg4::KanMX dot1::NatMX-GPDpr-5' UTR-TbDot1A -9xmyc-HphNt1	this study
NKI6148	MATa his3-1 leu2-0 lys2-0 met15d0 trp1d63 ura3-0 arg4::KanMX dot1::NatMX-ADHpr-TbDot1A-9xmyc-HphNt1	this study
NKI6149	MATa his3-1 leu2-0 lys2-0 met15d0 trp1d63 ura3-0 arg4::KanMX dot1::NatMX-TEFpr-TbDot1A-9xmyc-HphNt1	this study
NKI6150	MATa his3-1 leu2-0 lys2-0 met15d0 trp1d63 ura3-0 arg4::KanMX dot1::NatMX-GPDpr-TbDot1A-9xmyc-HphNt1	this study
NKI6080	MATa his3-1 leu2-0 lys2-0 met15d0 trp1d63 ura3-0 arg4::KanMX dot1::TbDot1B	this study
NKI6083	MATa his3-1 leu2-0 lys2-0 met15d0 trp1d63 ura3-0 arg4::KanMX dot1::NatMX-GPDpr-TbDot1B	this study
NKI6128	MATa his3-1 leu2-0 lys2-0 met15d0 trp1d63 ura3-0 arg4::KanMX dot1::NatMX-GPDpr-TbDot1B HMLα1/2Δ::HphMX	this study
NKI6085-1	MATa his3-1 leu2-0 lys2-0 met15d0 trp1d63 ura3-0 arg4::KanMX dot1::NatMX-TEFpr-TbDot1B	this study
NKI6129	MATa his3-1 leu2-0 lys2-0 met15d0 trp1d63 ura3-0 arg4::KanMX dot1::NatMX-TEFpr-TbDot1B HMLα1/2Δ::HphMX	this study
NKI6110	MATa his3-1 leu2-0 lys2-0 met15d0 trp1d63 ura3-0 arg4::KanMX dot1::NatMX-TEFpr-5'UTR-TbDot1B	this study
NKI6111	MATa his3-1 leu2-0 lys2-0 met15d0 trp1d63 ura3-0 arg4::KanMX dot1::NatMX-GPDpr-5'UTR-TbDot1B	this study
NKI6112	MATa his3-1 leu2-0 lys2-0 met15d0 trp1d63 ura3-0 arg4::KanMX dot1::NatMX-ADHpr-TbDot1B	this study
NKI6130-2	MATa his3-1 leu2-0 lys2-0 met15d0 trp1d63 ura3-0 arg4::KanMX dot1::TbDot1B-9xmyc-HphNt1	this study
NKI6131-2	MATa his3-1 leu2-0 lys2-0 met15d0 trp1d63 ura3-0 arg4::KanMX dot1::NatMX-TEFpr-5'UTR-TbDot1B-9xmyc-HphNt1	this study
NKI6132	MATa his3-1 leu2-0 lys2-0 met15d0 trp1d63 ura3-0 arg4::KanMX dot1::NatMX-GPDpr-5'UTR-TbDot1B-9xmyc-HphNt1	this study
NKI6133	MATa his3-1 leu2-0 lys2-0 met15d0 trp1d63 ura3-0 arg4::KanMX dot1::NatMX-ADHpr-TbDot1B-9xmyc-HphNt1	this study
NKI6134	MATa his3-1 leu2-0 lys2-0 met15d0 trp1d63 ura3-0 arg4::KanMX dot1::NatMX-TEFpr-TbDot1B-9xmyc-HphNt1	this study
NKI6135-2	MATa his3-1 leu2-0 lys2-0 met15d0 trp1d63 ura3-0 arg4::KanMX dot1::NatMX-GPDpr -TbDot1B-9xmyc-HphNt1	this study
NKI6164	MATa his3-1 leu2-0 lys2-0 met15d0 trp1d63 ura3-0 arg4::KanMX dot1::NatMX-GAL1pr-TbDot1B-9xmyc-HphNt1	this study
NKI6107	MATa his3-1 leu2-0 lys2-0 met15d0 trp1d63 ura3-0 arg4::KanMX dot1::NatMX-ADHpr-(tb)URA3	this study
NKI6120	MATa his3-1 leu2-0 lys2-0 met15d0 trp1d63 ura3-0 arg4::KanMX dot1::NatMX-ADHpr-hDot1L1-430	this study
NKI6126A	MATa his3-1 leu2-0 lys2-0 met15d0 trp1d63 ura3-0 arg4::KanMX dot1::hDot1L1-430-9xmyc-HphNT1	this study
NKI6123	MATa his3-1 leu2-0 lys2-0 met15d0 trp1d63 ura3-0 arg4::KanMX dot1::TRP1-GALpr-spacer-NLS-3xFLAG-hDot1L1-430-9xmyc-HphNT	this study
NKI3031	MATa leu2d0 lys2d0 ura3d0	De Vos et al, 2011
NKI6109	MATa leu2d0 lys2d0 ura3d0 NatMX-ADHpr-Dot1	this study
NKI6136-2	MATa leu2d0 lys2d0 ura3d0	this study
NKI6137	MATa leu2d0 lys2d0 ura3d0 KanMX-TEFpr-5'UTR-Dot1-9xmyc-HphNt1	this study
NKI6138	MATa leu2d0 lys2d0 ura3d0 KanMX-GPDpr-5'UTR-Dot1-9xmyc-HphNt1	this study
NKI6139	MATa leu2d0 lys2d0 ura3d0 KanMX-TEFpr-Dot1-9xmyc-HphNt1	this study
NKI6140	MATa leu2d0 lys2d0 ura3d0 KanMX-GPDpr-Dot1-9xmyc-HphNt1	this study
NKI8038	MATa leu2d0 lys2d0 ura3d0 KanMX-GPDpr-Dot1	this study
NKI8039	MATa leu2d0 lys2d0 ura3d0 KanMX-TEFpr-Dot1	this study
NKI8040	MATa leu2d0 lys2d0 ura3d0 KanMX-GPDpr-5'UTR-Dot1	this study
NKI8041	MATa leu2d0 lys2d0 ura3d0 KanMX-TEFpr-5'UTR-Dot1	this study
NKI2220	MATa his3d200 leu2d0 trp1d63 ura3d0 met15d0 hht1-hhf1::MET15 bar1::HisG HIS3 Pgpd_CRE_EBD78 hht2::HHT2-LoxP-T7-HYG-LoxP-HA+6xHis	Terweij et al, 2013
NKI8037	MATa his3d200 leu2d0 trp1d63 ura3d0 met15d0 hht1-hhf1::MET15 bar1::HisG HIS3 Pgpd_CRE_EBD78 hht2::HHT2-LoxP-HA+6xHis	Terweij et al, 2013
NKI4506	MATa his3d200 leu2d0 trp1d63 ura3d0 met15d0 hht1-hhf1::MET15 bar1::HisG HIS3 Pgpd_CRE_EBD78 hht2::HHT2-LoxP-T7-HYG-LoxP-HA+6xHis dot1::KanMX	this study
NKI6168	MATa his3d200 leu2d0 trp1d63 ura3d0 met15d0 hht1-hhf1::MET15 bar1::HisG HIS3 Pgpd_CRE_EBD78 hht2::HHT2-HYG-LoxP-HA+6xHis dot1::KanMX	this study
UCC7164	MATa ade2D::hisG his3d200 leu2d0 lys2d0 met15d0 trp1d63 ura3d0 ADE2-TEL-VR adh4::URA3-TEL (VII-L)	van Leeuwen, 2002
Y7092	MAT alpha can1d::STE2pr-Sp_his5 lyp1d his3d1 leu2d0 ura3d0 met15d0	Tong & Boone, 2006
NKI2298	MAT alpha can1d::STE2pr-Sp_his5 lyp1d his3d1 leu2d0 ura3d0 met15d0 dot1::GPDpr-yDOT1	this study
BY4726	MATalpha ade2d::hisG ura3d0	Brachman, 1998

Table SIIIA - H3K79 methyl dataset for computational simulations.

De Vos et al, 2011	H3K79me0	H3K79me1	H3K79me2	H3K79me3	copy number	Data from	Growth rate (k)
GAL1 pr_yDot1 0% gal	87	9	3	1	10	**MS**	0.29
GAL1 pr_yDot1 0,4% gal	48	27	16	8	100	**MS**	0.28
GAL1 pr_yDot1 0,8% gal	25	26	25	24	1000	**MS**	0.29
GAL1 pr_yDot1 1,4% gal	11	12	17	60	2000	**MS**	0.24

yDot1	H3K79me0	H3K79me1	H3K79me2	H3K79me3	copy number	Data from	Growth rate (k)
***DOT1* pr-yDot1**	**17**	**11**	**20**	**53**	**2000**	**MS**	0.39
GPD pr 5'UTR-yDot1	0	5	6	89	9043	wb	0.38
ADH pr-yDot1	4	4	4	88	16604	wb	0.37
***GPD* pr-yDot1**	**3**	**4**	**6**	**87**	**68154**	**MS**	0.36

TbDot1A	H3K79me0	H3K79me1	H3K79me2	H3K79me3	copy number	Data from	Growth rate (k)
DOT1 pr-TbDot1A	81	18	1	0	24	wb	0.37
TEF pr 5'UTR-TbDot1A	33	31	36	0	691	wb	0.37
***GPD* pr 5'UTR-TbDot1A**	**5**	**13**	**76**	**7**	**2122**	**MS**	0.37
***ADH* pr-TbDot1A**	**2**	**6**	**78**	**14**	**4417**	**MS**	0.36
***TEF* pr-TbDot1A**	**2**	**4**	**64**	**31**	**12059**	**MS**	0.36

TbDot1B	H3K79me0	H3K79me1	H3K79me2	H3K79me3	copy number	Data from	Growth rate (k)
DOT1 pr-TbDot1B	96	3	1	0	12	wb	0.38
***GAL1* pr-TbDot1B 1% gal**	**64**	**2**	**7**	**27**	**364**	**MS**	0.33
***TEF* pr 5'UTR-TbDot1B**	**35**	**2**	**9**	**54**	**1076**	**MS**	0.38
ADH pr-TbDot1B	0	0	2	97	4017	wb	0.38
***TEF* pr-TbDot1B**	**3**	**0**	**2**	**95**	**8402**	**MS**	0.38
***GPD* pr-TbDot1B**	**3**	**0**	**2**	**95**	**13955**	**MS**	0.35

hDot1L 1-430	H3K79me0	H3K79me1	H3K79me2 home made	H3K79me3	copy number	Data from	Growth rate (k)
GAL1 pr_hDot1L 1-430 0% gal	100	1	0	0	240	wb	0.35
***GAL1* pr_hDot1L 1-430 2% gal**	**89**	**11**	**0**	**0**	**5198**	**MS**	0.22
**2μ *GAL1* pr_hDot1L 1-430 2% gal**	**45**	**13**	**42**	**0**	**1053397**	**MS**	0.22

Table SIIIB-D present the subsequent steps to build this table.

Table SIIIB - H3K79 methyl signals from western blots + SEM

	Average western blot signals					SEM western blot signals			
**yDot1**	**H3K79me0**	**H3K79me1**	**H3K79me2**	**H3K79me3**	**copy number**	**H3K79me1**	**H3K79me2**	**H3K79me3**	**copy number**
TEF pr 5'UTR-yDot1	16	12	16	57	2000	0.39	0.02	0.00	606
DOT1 pr-yDot1	6	10	14	70	1058	0.35	0.33	0.04	207
GPD pr 5'UTR-yDot1	-2	6	7	89	9043	0.22	0.16	0.15	2566
ADH pr-yDot1	4	4	4	88	16604	0.21	0.06	0.18	3305
TEF pr-yDot1	16	5	3	77	27945	0.18	0.08	0.02	12902
GPD pr-yDot1	-5	5	4	96	68154	0.06	0.01	0.34	32112

**TbDot1A**	**H3K79me0**	**H3K79me1**	**H3K79me2**	**H3K79me3**	**copy number**	**H3K79me1**	**H3K79me2**	**H3K79me3**	**copy number**
DOT1 pr-TbDot1A	88	18	1	-6	24	0.60	0.02	0.02	1
TEF pr 5'UTR-TbDot1A	33	31	36	-1	691	0.09	0.42	0.06	22
GPD pr 5'UTR-TbDot1A	10	11	70	8	2122	0.03	0.47	0.03	112
ADH pr-TbDot1A	8	4	78	10	4417	0.01	0.10	0.04	95
TEF pr-TbDot1A	-6	2	71	33	12059	0.01	0.18	0.13	-
GPD pr-TbDot1A	12	1	53	35	15414	0.01	0.51	0.10	-

**TbDot1B**	**H3K79me0**	**H3K79me1**	**H3K79me2**	**H3K79me3**	**copy number**	**H3K79me1**	**H3K79me2**	**H3K79me3**	**copy number**
DOT1 pr-TbDot1B	101	3	1	-5	12	0.20	0.02	0.02	2
GAL1 pr-TbDot1B 1% gal	68	2	4	27	364	0.04	0.04	0.15	183
TEF pr 5'UTR-TbDot1B	13	1	8	79	1076	0.04	0.20	0.16	310
GPD pr 5'UTR-TbDot1B	-10	0	2	108	3709	0.03	0.01	0.42	1334
ADH pr-TbDot1B	-3	0	2	100	4017	0.04	0.03	0.28	926
TEF pr-TbDot1B	-1	0	5	95	8402	0.02	0.28	0.13	889
GPD pr-TbDot1B	3	0	4	92	13955	0.01	0.23	0.19	-

**hDot1L 1-430**	**H3K79me0**	**H3K79me1**	**H3K79me2 home made**	**H3K79me3**	**copy number**	**H3K79me1**	**H3K79me2 home made**	**H3K79me3**	**copy number**
GAL1 pr_hDot1L 1-430 0% gal	100	1	0	0	240	0.04	0.00	0.00	89
GAL1 pr_hDot1L 1-430 2% gal	85	18	1	-3	5198	11.71	0.15	0.36	726
2µ *GAL1* pr_hDot1L 1-430 2% gal	63	23	16	-2	1053397	4.54	2.68	0.43	378664

Table SIIIC - MS data + SEM

	Average MS values				SEM MS values			
**MS_averages**	**H3K79me0**	**H3K79me1**	**H3K79me2**	**H3K79me3**	**H3K79me0**	**H3K79me1**	**H3K79me2**	**H3K79me3**
DOT1 pr-yDot1	17	11	20	53	0.7	0.0	0.0	1.4
GPD pr-yDot1	3	4	6	87	0.0	0.0	0.0	0.0
GPD pr 5'UTR-TbDot1A	5	13	76	7	1.4	0.7	2.8	1.4
ADH pr-TbDot1A	2	6	78	14	0.0	0.0	1.4	2.1
TEF pr-TbDot1A	2	4	64	31	0.7	0.7	0.0	0.0
GAL1 pr-TbDot1B 1% gal	64	2	7	27	4.7	0.0	0.4	5.1
TEF pr 5'UTR-TbDot1B	35	2	9	54	0.2	0.1	0.5	0.2
TEF pr-TbDot1B	3	0	2	95	0.0	0.0	0.0	0.0
GPD pr-TbDot1B	3	0	2	95	0.0	0.0	0.0	0.0
GAL1 pr_hDot1L 1-430 2% gal	89	11	0	0	2.1	2.0	0.2	0.1
2µ *GAL1* pr_hDot1L 1-430 2% gal	45	13	42	0	2.0	1.5	0.6	0.0

Table SIIID - Integrated dataset with quality control.

yDot1	H3K79me0	H3K79me1	H3K79me2	H3K79me3	copy number	Data from	total methyl number	Change (%)	Growth rate (k)	Note
TEF pr 5'UTR-yDot1	6	10	14	70	1058	wb	247		0.39	OUTLIER; deviates from the reference.
DOT1 pr-yDot1	17	11	20	53	2000	MS	210	-18	0.39	**REFERENCE**
GPD pr 5'UTR-yDot1	0	5	6	89	9043	wb	285	26	0.38	Negative value for H3K79me0 was set to zero and 1% was added to H3K79me1 and H3K79me2.
ADH pr-yDot1	4	4	4	88	16604	wb	276	-3	0.37	
TEF pr-yDot1	16	5	3	77	27945	wb	240	-15	0.37	OUTLIER
GPD pr-yDot1	3	4	6	87	68154	MS	277	13	0.36	

TbDot1A	H3K79me0	H3K79me1	H3K79me2	H3K79me3	copy number	Data from	total methyl number	Change (%)	Growth rate (k)	Note
DOT1 pr-TbDot1A	81	18	1	0	24	wb	19		0.37	
TEF pr 5'UTR-TbDot1A	33	31	36	0	691	wb	104	82	0.37	
GPD pr 5'UTR-TbDot1A	5	13	76	7	2122	MS	186	44	0.37	
ADH pr-TbDot1A	2	6	78	14	4417	MS	203	8	0.36	
TEF pr-TbDot1A	2	4	64	31	12059	MS	225	10	0.36	
GPD pr-TbDot1A	12	1	53	35	15414	wb	211	-6	0.35	OUTLIER

TbDot1B	H3K79me0	H3K79me1	H3K79me2	H3K79me3	copy number	Data from	total methyl number	Change (%)	Growth rate (k)	Note
DOT1 pr-TbDot1B	96	3	1	0	12	wb	4		0.38	
GAL1 pr-TbDot1B 1% gal	64	2	7	27	364	MS	96	96	0.36	
TEF pr 5'UTR-TbDot1B	35	2	9	54	1076	MS	184	48	0.38	
GPD pr 5'UTR-TbDot1B	-10	0	2	108	3709	wb	329	44	0.38	OUTLIER
ADH pr-TbDot1B	0	0	2	97	4017	wb	296	38	0.38	100% H3K79me3 was corrected to 97% to avoid a negative value for H3K79me0
TEF pr-TbDot1B	3	0	2	95	8402	MS	289	-2	0.38	
GPD pr-TbDot1B	3	0	2	95	13955	MS	289	0	0.35	

hDot1L 1-430	H3K79me0	H3K79me1	H3K79me2 home made	H3K79me3	copy number	Data from	total methyl number	Change (%)	Growth rate (k)	Note
GAL1 pr_hDot1L 1-430 0% gal	100	1	0	0	240	wb	0		0.35	
GAL1 pr_hDot1L 1-430 2% gal	89	11	0	0	5198	MS	12		0.22	
2μ *GAL1* pr_hDot1L 1-430 2% gal	45	13	42	0	1053397	MS	97	88	0.22	

Table SIV. Predicted rate constants ($\mu M^{-1}min^{-1}$) and errors for Dot1 enzymes.

H3K79me	k_x	yDot1*	TbDot1A	TbDot1B
H3K79me0				
	k_0	0.049 (0.0052)	0.047 (0.0063)	0.018 (0.0013)
H3K79me1				
	k_1	0.021 (0.0017)	0.022 (0.0015)	0.30 (0.070)
H3K79me2				
	k_2	0.010 (0.00061)	0.00036 (0.000029)	0.080 (0.016)
H3K79me3				
SS		0.17 (0.039)	0.081 (0.019)	0.086 (0.028)

Supplemental Methods

H3K79 methylation pattern analysis

For analysis of H3K79 methylation and Dot1 protein expression cells were harvested in mid-log phase. Proteins were extracted using the SUMEB protocol [5]. H3K79 methylation signals were detected using home-made antibodies against H3K79me1, -me2 and -me3 [1] and H3K79me2 (04-835, Millipore), while antibodies for histone H3 [1] and Pgk1 (A-6457, Invitrogen) were used as loading controls. To determine Dot1 copy numbers TbDot1A and TbDot1B were C-terminally 9xMyc-tagged as described above and expression levels were compared to yDot1-9xMyc expression levels using a Myc antibody (sc-789, Santa Cruz). H3K79 methylation patterns were identical for TbDot1A and -B with and without 9xMyc tag as expected [6]. yDot1 expression levels were detected using a yDot1 home-made antibody [7]. hDot1L copy numbers in yeast were estimated using the 9xMyc tag and 3xFLAG tag detected with Flag-M2 (F3165, Sigma). As the full length hDot1L enzyme was not more active than the shorter variant (lacking the non-conserved C-terminal domain), the shorter hDot1L 1-430 variant was used in this study (results not shown and [8]). Quantitative western blot analysis was performed using the LI-COR Odyssey IRDye® IR imager (Biosciences) and the Odyssey LI-COR software. For details about the normalization of the H3K79 methylation signals based on the linearity of the antibodies, see below. Histone H3 purification for mass spectrometry and determination of growth rates was performed as described previously [1]. Data were obtained of at least two replicates.

H3K79 methylation signal normalization and antibody validation

To determine H3 antibody specificity, H3 was detected in a dilution series of a yeast protein extract. This revealed that our H3 antibody [1] does not have a linear detection range but showed the relationship $y=x^{0.5}$, in which x is the measured H3 signal and y the corrected signal. The relationship was used to normalize the H3 immunoblot signals on each blot. H3K79 methylation signals were normalized over H3. To validate the linearity of the H3K79 methylation antibodies, quantitative western blot signals were plotted against absolute H3K79 methylation levels determined by mass spectrometry (Figure 1C-D). In GraphPad Prism 6.0 software, a non-linear regression fit was applied using the option 'straight line'. The correlation between western blot and MS data for the H3K79me1 and –me3 home-made antibodies and the H3K79me2 antibody from Millipore was good based on the R squares, and these antibodies were used for the analysis described in this study. The H3K79 methylation signals were normalized using the polynomial function that resulted from the correlation analysis (H3K79me1 $y=0.084x$; H3K79me2 (MP) $y=0.064x$; H3K79me3 $y=0.016x+0.061$ in which x and y are respectively the detected and normalized H3K79 methylation signals). To detect the H3K79me2 profile generated by hDot1L, the H3K79me2 homo made antibody was used as the non-linearity is caused by cross-reactivity with H3K79me3, which is not generated by hDot1L.

H3K79 methylation, Dot1 expression and growth rates for simulation.

For computational simulations, the H3K79 methylation data from quantitative western-blots (Table SIIIB) were replaced by MS data (Table SIIIC) when available, to generate a high quality quantitative dataset (Table SIIID). Since western-blot data are semi-quantitative, we put a few constrains to the data to select only the most reliable data for modeling purposes (Table SIIIA).

First, negative values for H3K79 methylation were set to zero (H3K79me0 drops as a consequence). Second, a quality check was performed to determine whether individual strains behaved as outliers. Therefore, the total methyl number (TMN) was calculated (maximum is 100% H3K79me3, which would be TMN=300). Per Dot1 enzyme, strains were ranked in order of increasing Dot1 copy number. When the TMN dropped >10% compared to the preceding strain or when TMN>330, strains were considered as outliers and removed from the dataset (Table SIIIA). Growth rates of strains expressing low and high levels of each Dot1 protein were determined by cell counting. Growth rates of strains expressing intermediate levels of each Dot1 protein were estimated based on relative colony growth of the strains compared to strains with low ahd high expression of the respective Dot1 protein.

Computational simulation of H3K79 methylation patterns.

We made use of a kinetic model reported previously to predict experimental H3K79 methylation data for the distributive yeast Dot1 enzyme from *S. cerevisiae* [9]. The model described a basic distributive enzyme mechanism in combination with constant histone production (γ) and first order growth dilution of all histones (specific growth rate μ) as a result of the cell volume increase [10,11]. The state variables corresponding to the nuclear concentrations of unmethylated H3K79 (H3K79me0), mono- (H3K79me1), di- (H3K79me2) and trimethylated (H3K79me3) were described by differential equations, respectively:

$$\begin{cases} \frac{d(me0(t))}{dt} = \gamma - \mu \cdot me0(t) - v_0 \\ \frac{d(me1(t))}{dt} = -\mu \cdot me1(t) + v_0 - v_1 \\ \frac{d(me2(t))}{dt} = -\mu \cdot me2(t) + v_1 - v_2 \\ \frac{d(me3(t))}{dt} = -\mu \cdot me3(t) + v_2 \end{cases}$$

with sequential methylation steps represented by mass-action kinetics.

$$\begin{cases} v_0 = k_0 \cdot dot \cdot me0(t) \\ v_1 = k_1 \cdot dot \cdot me1(t) \\ v_2 = k_2 \cdot dot \cdot me2(t) \end{cases}$$

The proportionality with the Dot1 concentration ('dot' in the equations) is in accordance with the assumption that the enzyme (Dot1) levels are significantly smaller than the protein substrate H3 levels. The Dot1 concentration is determined by the estimations from immunoblot experiments. As previous demonstrated [9], more complicated enzyme kinetic equations (describing substrate saturation or competition or product inhibition) have typically too many parameters to allow a unique fit to the available experimental data. Moreover, the Dot1 substrate S-adenosylmethionine (AdoMet) was assumed to be constant. The kinetic constants in this study therefore represent apparent rate constants. The constant γ is set by the steady state assumption that histone production equals loss by growth dilution, *i.e..* $\gamma = \mu$ *(*me0* + *me1* + *me2* + *me3*) = μ *H_{tot}. This assumption disregards the possible involvement of replication-independent histone turn-over in yeast [12–15] and may lead to an underestimation of the histone production and the rate constants. Whereas further model extensions may be possible in the future, the

experimental validation in this study and De Vos et al. [9] have nevertheless affirmed the predictive power of our current approach.
To evaluate whether the methylation data agree more strongly with either a distributive or a processive enzyme mechanism the following basic model was used (see also [9]). It describes a processive enzyme mechanism with the unmethylated histone as only substrate and the subsequent methylated forms as (leaky) intermediates or products and was defined as follows:

$$\begin{cases} \frac{d(me0(t))}{dt} = \gamma - \mu \cdot me0(t) - v_0 - v_{0'} - v_{0''} \\ \frac{d(me1(t))}{dt} = - \mu \cdot me1(t) + v_0 \\ \frac{d(me2(t))}{dt} = - \mu \cdot me2(t) + v_{0'} \\ \frac{d(me3(t))}{dt} = - \mu \cdot me3(t) + v_{0''} \end{cases},$$

$$\begin{cases} v_0 = k_0 \cdot dot \cdot me0(t) \\ v_{0'} = k_{0'} \cdot dot \cdot me0(t) \\ v_{0''} = k_{0''} \cdot dot \cdot me0(t) \end{cases}.$$

In this mechanism the unmethylated H3K79 yields either the trimethylated form, without release from the enzyme, or leads to mono- or di-methylated H3K79 intermediates through irreversible dissociation from Dot1 (hence it leakiness). Like for the distributive model the enzyme activity is represented by three mass-action kinetic equations (v_0's) with corresponding rate constants (k_0's). The other parameters are equivalent between the models enabling straight-forward comparison and justifying our conclusion that the processive model was generally not as successful in simulating the experimental data for all enzymes analyzed.

The comparative test to estimate the rate constants consisted of a parameter estimation by ordinary least squares optimization using non-linear regression according to the procedure described [9]. The input from experiments consisted of the fractions of the different methylation states for different growth rates and Dot1 concentrations (Table SIII) and the simulation output determining the objective function to be minimized consisted of the predicted unique steady-state methylation fractions. The values for the Dot1 concentration (*dot*), the specific growth rate (μ), and the histone synthesis rate (γ, depending on H_{tot} as explained above) were considered to be fixed in this procedure. The amounts of histone H3 was taken to be 200,000 molecules/cell [16]. These values were converted into concentrations using an approximate cell nucleus volume of 2.9 µm^3 in wild type cells grown in glucose media [17]. Briefly, numerical integration runs via the NDSolve function of Mathematica™ (7.0) (http://www.wolfram.com/) were used to evaluate the least squares objective function as a function of the Dot1 kinetic constants with the NMinimize function with the 'Automatic' search method within realistic parameter bounds (on a MacBook with Intel Core i7 CPU). The sum of the squares (SS) is a direct measure of the accuracy of the fit for a specific dataset (as also can be visually assessed via the corresponding prediction of the methylation states). Moreover, the influence of data

uncertainty on the resulting parameter estimates was investigated via a Monte Carlo-type approach with multiple estimation runs (until convergence of the mean parameter estimates) with artificial Gaussian noise (amounting to a mean coefficient of variation of 10 % superposed on the data, yielding parameter error estimates (Table SIV). First, the distributive model was evaluated against an extended dataset from yeast (Table SIIIE) with resulted in parameter estimates very close to the original ones (Table I). The improved parameter values will submitted to the JWS database (jjj.biochem.sun.ac.za/database/) upon publication to update the deposited model.
Then the presented distributive and processive models were used to simulate the H3K79 methylation patterns determined by western blot and mass spectrometry of TbDot1A, TbDot1B and hDot1L. For hDot1L, in the absence of any significant trimethylated fraction, the trimethylation reaction rate constant could not be determined and was set to zero. The experimental estimate of the hDot1L for the highest copy number data point (Fig. S4, middle panel) led to an underestimation of the methylation fractions of the second data point. This indicated that the very high copy number did not represent an equivalent enzyme activity level (see Results). Simultaneously estimating the copy number for the third data point resolved this problem (Fig. S4). The distributive model successfully fitted the experimental data of yDot1, TbDot1A, TbDot1B and hDot1L.

Single cell models for methylation dynamics during the cell cycle
A model was constructed to describe dynamic changes in histone methylation during the Trypanosoma cell cycle. This model accounts for one cell cycle of 13 hours [2] in which the G2/M, S and G1 phases take up respectively 40%, 20% and 40% of the time [3]. Histone synthesis was assumed to take place strictly in S phase [15], whereas growth dilution is assumed to be continuous [10,11]. The value of the production rate (γ) was set such that the value averaged over the complete cell cycle equals the growth dilution rate. No explicit cell division was taken into account assuming that this does not affect the nuclear concentrations. Starting from a random set of initial conditions the model was simulated and the final methylation states replaced the initial values until starting and final states matched close enough (within 0.1% methylation) in order to represent a stable cycle. The values from the parameter estimation runs were used for the reaction rate constants (Table I). To establish a model for H3K76 methylation during the trypanosome cell cycle, TbDot1A and –B expression levels and activity window were manually fit to recapitulate the published H3K76 methylation pattern in trypanosomes (see below). The total histone concentration was taken to be 840,000 molecules/cell (based on dividing the (diploid) genome of 70 Mb by 166 bp/nucleosome or 170 µM for an approximate nuclear volume of 8.2 μm^3 (based on (Daniels et al, 2010; MMBR)). Subsequently, TbDot1A and –B conditions were varied to reproduce reported H3K76 methylation patterns under varying conditions [2,4].

H3K76 methylation states in trypanosomes described in literature and assumptions for modeling
<u>Literature:</u>
- No H3K76me1 or H3K76me2 is observed during S-phase [4].
- H3K76me1 is observed mainly in G2 and M phase cells [4].

- The percentage of cells in which H3K76me1 is detected does not decrease much from G2/M to cytokinesis [4].
- H3K76me1 is observed in twice as many cells as H3K76me2 during G2/M [4].
- H3K76me2 is observed from early mitosis onwards [2].
- H3K76me2 is observed in approximately twice as many cells as H3K76me1 [4] during cytokinesis.
- A few cells have H3K76me1 or –me2 [4].
- H3K76me3 is detected during all stages of the cell cycle [2].

Assumptions:

1. TbDot1B is only expressed in G1-phase:
   a. TbDot1B is not expressed during G2/M. If TbDot1B would be expressed in G2/M, it would efficiently convert the H3K76me1 and –me2 signals into H3K76me3, leaving no H3K76me1 and –me2 signals to be detected.
   b. TbDot1B is not expressed during S-phase. If TbDot1B would be expressed in S-phase, it would efficiently put H3K76me3 on the newly synthesized unmethylated histones H3, leaving no histones that can become mono- or dimethylated in G2/M.
2. TbDot1A is expressed in G2/M and G1-phase:
   a. TbDot1A is not expressed in S-phase as no H3K76me1 or –me2 signals are observed during this stage of the cell cycle.
   b. TbDot1A is expressed in G2/M since only TbDot1A is able to generate the consecutive peaks of H3K76me1 and –me2.
3. No H3K79me0 remains at the G2/M to G1 transition:
   a. As TbDot1B has to convert all lower H3K76 methylation states to H3K76me3 rapidly, we assume that all H3K76me0 is converted to H3K76me1 by TbDot1A at the start of G1 as TbDot1B has a low rate constant for the conversion to H3K76me1 (*k0*).
   b. Additional simulations with reduced TbDot1A copies leaving some residual H3K76me0 at the start of the G2/M to G1 transition resulted in a peak of H3K76me2 at the start of G1 (as also displayed in Figure 5E), which has never been observed in trypanosomes.
4. H3K76me1 peaks during G2/M-phase:
   a. More G2/M than cells in cytokinesis were stained for H3K76me1, which indicates that the H3K76me1 peaks before cytokinesis.
5. H3K76me2 peaks at the end of mitosis:
   a. During cytokinesis, almost every cell displayed H3K76me2 staining.

Supplemental References

1. Frederiks, F. *et al.* Nonprocessive methylation by Dot1 leads to functional redundancy of histone H3K79 methylation states. *Nat. Struct. Mol. Biol.* **15,** 550–7 (2008).

2. Janzen, C. J., Hake, S. B., Lowell, J. E. & Cross, G. A. M. Selective di- or trimethylation of histone H3 lysine 76 by two DOT1 homologs is important for cell cycle regulation in *Trypanosoma brucei*. *Mol. Cell* **23,** 497–507 (2006).

3. McKean, P. G. Coordination of cell cycle and cytokinesis in *Trypanosoma brucei*. *Curr. Opin. Microbiol.* **6,** 600–607 (2003).

4. Gassen, A. *et al.* DOT1A-dependent H3K76 methylation is required for replication regulation in *Trypanosoma brucei*. *Nucleic Acids Res.* **40,** 10302–11 (2012).

5. Gardner, R. *et al.* Sequence determinants for regulated degradation of yeast 3-hydroxy-3-methylglutaryl-CoA reductase, an integral endoplasmic reticulum membrane protein. *Mol. Biol. Cell* **9,** 2611–26 (1998).

6. Frederiks, F. *et al.* Heterologous expression reveals distinct enzymatic activities of two DOT1 histone methyltransferases of Trypanosoma brucei. *J. Cell Sci.* **123,** 4019–23 (2010).

7. Van Leeuwen, F., Gafken, P. R. & Gottschling, D. E. Dot1p modulates silencing in yeast by methylation of the nucleosome core. *Cell* **109,** 745–56 (2002).

8. Deshpande, A. J. *et al.* AF10 Regulates Progressive H3K79 Methylation and HOX Gene Expression in Diverse AML Subtypes. *Cancer Cell* **26,** 896–908 (2014).

9. De Vos, D. *et al.* Progressive methylation of ageing histones by Dot1 functions as a timer. *EMBO Rep.* **12,** 956–62 (2011).

10. Goranov, A. I. *et al.* The rate of cell growth is governed by cell cycle stage. *Genes Dev.* **23,** 1408–22 (2009).

11. Bryan, A. K., Goranov, A., Amon, A. & Manalis, S. R. Measurement of mass, density, and volume during the cell cycle of yeast. *Proc. Natl. Acad. Sci. U. S. A.* **107,** 999–1004 (2010).

12. Dion, M. F. *et al.* Dynamics of replication-independent histone turnover in budding yeast. *Science* **315,** 1405–8 (2007).

13. Jamai, A., Imoberdorf, R. M. & Strubin, M. Continuous histone H2B and transcription-dependent histone H3 exchange in yeast cells outside of replication. *Mol. Cell* **25,** 345–55 (2007).

14. Rufiange, A., Jacques, P.-E., Bhat, W., Robert, F. & Nourani, A. Genome-wide replication-independent histone H3 exchange occurs predominantly at promoters and implicates H3 K56 acetylation and Asf1. *Mol. Cell* **27,** 393–405 (2007).

15. Verzijlbergen, K. F. *et al.* Recombination-induced tag exchange to track old and new proteins. *Proc. Natl. Acad. Sci. U. S. A.* **107,** 64–8 (2010).

16. Ghaemmaghami, S. *et al.* Global analysis of protein expression in yeast. *Nature* **425,** 737–41 (2003).

17. Jorgensen, P. *et al.* The size of the nucleus increases as yeast cells grow. *Mol. Biol. Cell* **18,** 3523–32 (2007).
